# Supplementary material for: Type I interferon signaling in hematopoietic cells impairs neutrophil antibacterial function in the middle ear during viral co-infection
Source: Cell Rep Med. 2026 Jun 3;7(6):102846. doi: 10.1016/j.xcrm.2026.102846 (PMC13293972; doi:10.1016/j.xcrm.2026.102846)
Supplement: Document S1. Figures S1–S6 [file mmc1.pdf]

Cell Reports Medicine, Volume *7*

Supplemental information

Type I interferon signaling in hematopoietic cells impairs neutrophil antibacterial function in the middle ear during viral co-infection

Steven C. Shaw, Taylor L. Jamil, Gabriela Heslop, Jeremy T. Fleck, Wyatt Johnson, Brian P. Lorenz, Zoe Drigot, J. Kirk Harris, Sarah A. Gitomer, and Sarah E. Clark

Supplementary Information

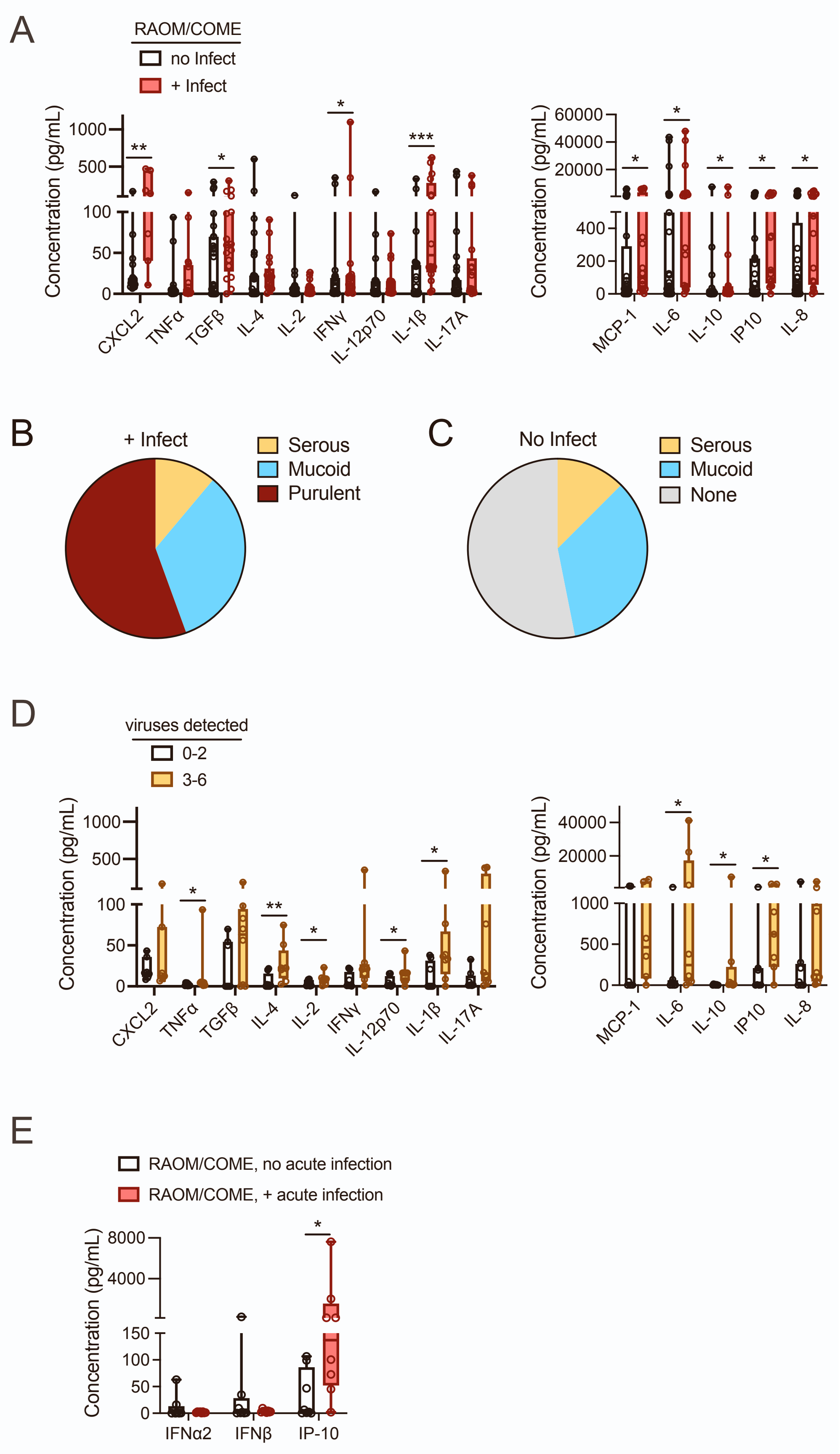
A
RAOM/COME
no Infect
+ Infect
Concentration (pg/mL)
CXCL2
TNFα
TGFβ
IL-4
IL-2
IFNγ
IL-12p70
IL-1β
IL-17A
MCP-1
IL-6
IL-10
IP10
IL-8
B
+ Infect
Serous
Mucoid
Purulent
C
No Infect
Serous
Mucoid
None
D
viruses detected
0-2
3-6
E
RAOM/COME, no acute infection
RAOM/COME, + acute infection
IFNα2
IFNβ
IP-10

Supplementary Figure 1 (related to Figure 1). Middle ear effusion characteristics. (A) Concentration of inflammatory cytokines and chemokines detected in middle ear fluid samples from children with rAOM or COME with a clinical diagnosis of acute OM (+ Infect, n=18 subjects) or no diagnosis of acute OM (no Infect, n=31 subjects), individual values plotted from Figure 1C. (B-C) Percentage of patient samples with middle ear effusions detected, with effusions classified as serous, mucoid, or purulent, among children with rAOM or COME with a clinical diagnosis of active OM (+ Infect, n=18 subjects), (B) or no clinical diagnosis of active OM (No Infect, n=31 subjects), (C). (D) Concentration of inflammatory cytokines and chemokines detected in middle ear fluid samples from children with rAOM or COME with 0-2 individual viruses detected (n=8 subjects) or 3-6 viruses detected (n=8 subjects), from Figure 1D. (E) Concentration of IFNα2, IFNβ, and IP-10 (CXCL10) detected in middle ear fluid samples from children with rAOM or COME with a clinical diagnosis of acute OM (+ Infect, n=8 subjects) or no diagnosis of acute OM (no Infect, n=8 subjects), detected in the subset of available samples from (A). Samples at or below the limit of detection are reported as 0 pg/mL. Box boundaries indicate the 25th and 75th percentiles, with a horizontal line representing the median and whiskers indicating minimum and maximum values. *p<.05, **p<.01, ***p<.001, Mann-Whitney U test.

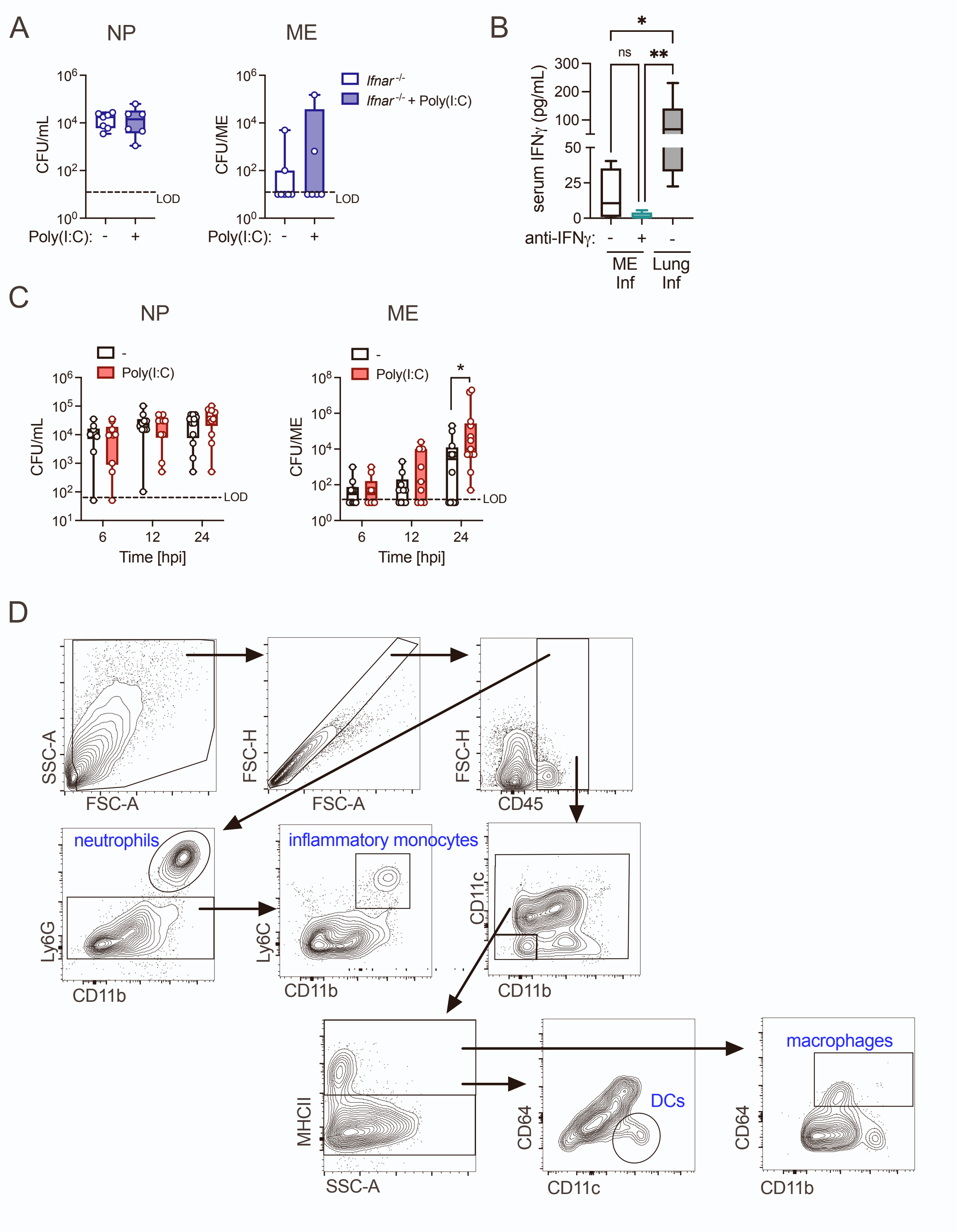

Supplementary Figure 2 (related to Figures 3-4). IFNγ depletion and middle ear infection time course. (A) Burden of *S. pneumoniae* serotype 7F detected in the nasopharynx (NP) and middle ear (ME) of *Ifnar*$^{-/-}$ mice at 24

hours post-infection (10^5 CFU/mouse i.n.) with or without co-treatment with poly(I:C) (50 µg/mouse i.n.), n=6-7 mice/grp. (B) Concentration of IFNγ detected in the serum of WT mice infected with *S. pneumoniae* intranasally (10^5 CFU/mouse i.n.) to induce middle ear (ME) infection or intratracheally ($5x10^6$ CFU/mouse i.t.) to induce lung infection 24 hours following treatment with anti-IFNγ antibody or isotype control antibody (200 µg/mL i.p.), n=7-9 mice/grp. (C) Burden of *S. pneumoniae* detected at indicated time points post-infection (10^5 CFU/mouse i.n.) with or without co-treatment with poly(I:C), n= 10 mice/grp (6 h, 12 h), n= 19 mice/grp (24 h). (D) Gating strategy to detect neutrophils ($CD45^+Ly6G^+CD11b^+$), inflammatory monocytes ($CD45^+Ly6G^-Ly6C^+CD11b^+$), dendritic cells (DCs, $CD45^+MHCII^+CD64^-CD11c^+$), and macrophages ($CD45^+CD64^+CD11b^+$) in middle ear samples by flow cytometry. Data pooled from 2 (A) or 3 (B, C) independent experiments. Box boundaries indicate the 25th and 75th percentiles, with a horizontal line representing the median and whiskers indicating minimum and maximum values. LOD = limit of detection. *$p<.05$, **$p<.01$, one-way ANOVA with Tukey's post hoc test (B), Mann-Whitney U test (C).

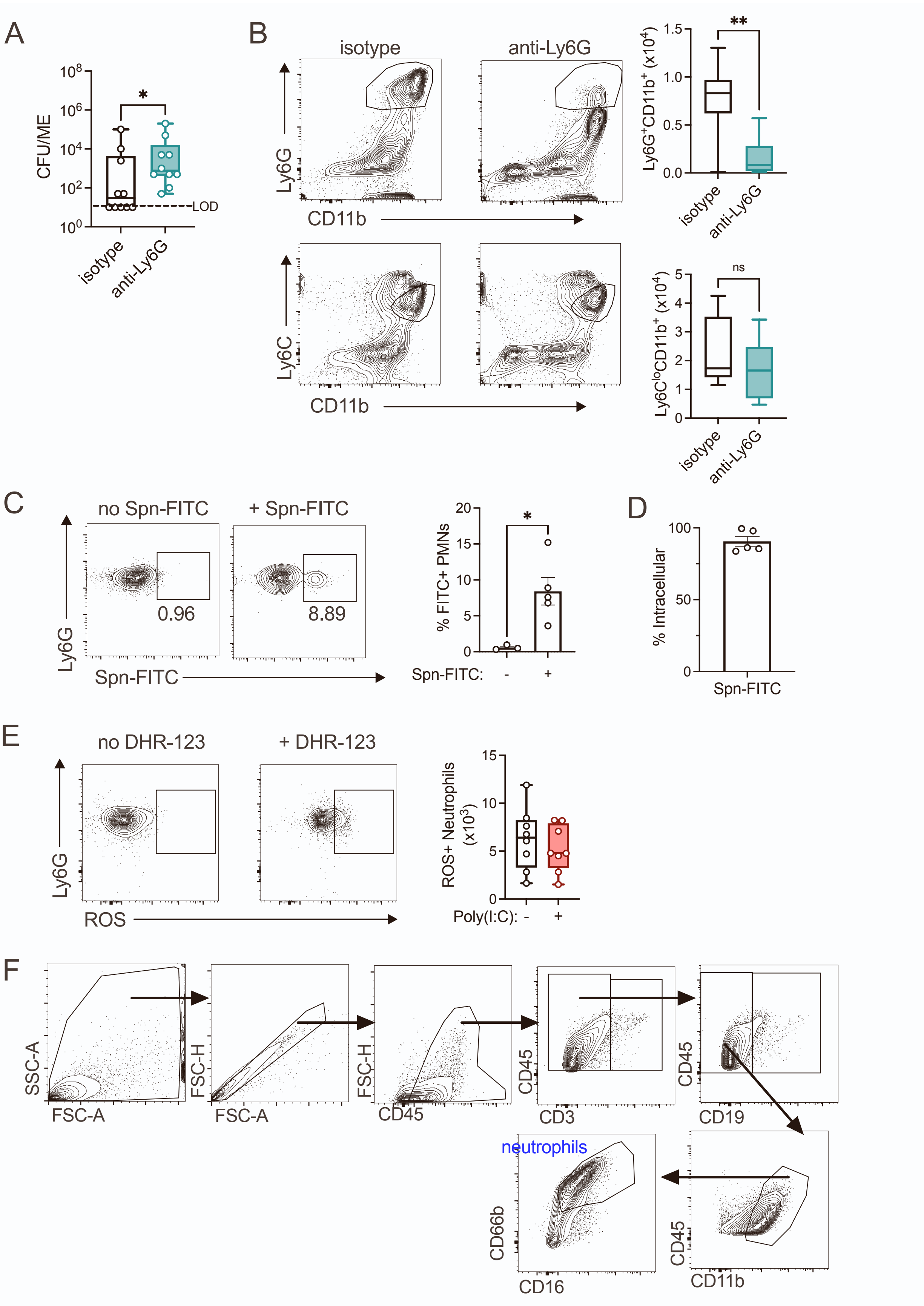
A
CFU/ME
10^8
10^6
10^4
10^2
10^0
*
LOD
isotype
anti-Ly6G
B
isotype
anti-Ly6G
Ly6G
CD11b
Ly6C
CD11b
Ly6G+CD11b+ (x10^4)
**
1.5
1.0
0.5
0.0
isotype
anti-Ly6G
Ly6C^loCD11b+ (x10^4)
ns
5
4
3
2
1
0
isotype
anti-Ly6G
C
no Spn-FITC
+ Spn-FITC
0.96
8.89
Ly6G
Spn-FITC
% FITC+ PMNs
20
15
10
5
0
*
Spn-FITC: - +
D
% Intracellular
100
50
0
Spn-FITC
E
no DHR-123
+ DHR-123
Ly6G
ROS
ROS+ Neutrophils (x10^3)
15
10
5
0
Poly(I:C): - +
F
SSC-A
FSC-A
FSC-H
FSC-A
FSC-H
CD45
CD45
CD3
CD45
CD19
neutrophils
CD66b
CD16
CD45
CD11b

Supplementary Figure 3 (related to Figure 5). Neutrophil depletion and detection of neutrophil phagocytosis and ROS. (A) Burden of *S. pneumoniae* serotype 7F detected in the middle ear of WT mice at 24 hours post-infection (10^5 CFU/mouse i.n.) in mice treated 24 hours prior to infection with anti-Ly6G antibody or isotype control antibody (200 µg/mL i.p.), n=10 mice/grp. (B) Representative flow cytometry plots and total number of $Ly6G^+CD11b^+$ gated neutrophils and $Ly6C^{lo}CD11b^+$ gated neutrophils in mice from (A). (C) Representative flow cytometry plots and percentage of Spn-$FITC^+$ neutrophils detected following 1 hour incubation with or without FITC-labeled heat-killed *S. pneumoniae*, for neutrophils detected in the blood of WT naïve mice, n=3-5 mice/grp. (D) Percentage of intracellular *S. pneumoniae* detected by Spn-FITC uptake assay. (E) Representative flow cytometry plots and total number of ROS+ neutrophils detected with the DHR-123 probe by flow cytometry in the middle ear of WT mice at 24 hours post-infection with *S. pneumoniae* (10^5 CFU/mouse i.n.) with or without co-treatment with poly(I:C) (50 µg/mouse i.n.), n=8 mice/grp. (F) Gating strategy to detect human neutrophils ($CD45^+CD3^-CD19^-CD11b^+CD66b^+CD16^+$) from blood and middle ear samples, with representative plots from a blood sample. Data pooled from 2 (C, D) or 3 (A, B, E) independent experiments. Box boundaries indicate the 25th and 75th percentiles, with a horizontal line representing the median and whiskers indicating minimum and maximum values. LOD = limit of detection. *p<.05, Mann-Whitney U test (A, B), unpaired t test (C).

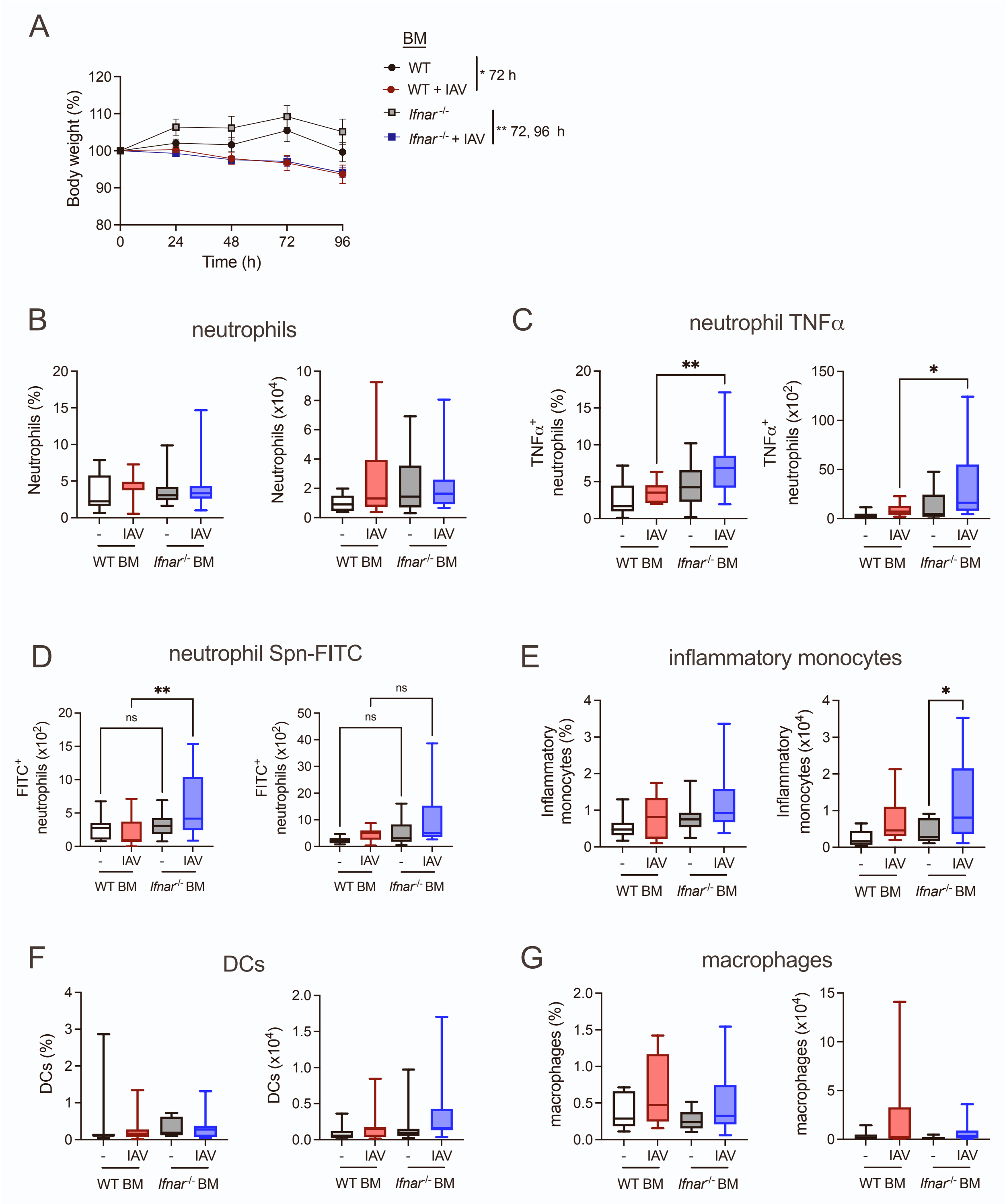

Supplementary Figure 4 (related to Figure 6). Impact of IFNAR signaling in irradiation-sensitive cells on immune cell recruitment and activation in the middle ear during *S. pneumoniae* serotype 2 infection. (A) Percent of starting body weight over time in irradiated WT recipients of WT or *Ifnar*$^{-/-}$ bone marrow with or without IAV co-infection (10^5 PFU/mouse i.n.) 72 hours prior to challenge with *S. pneumoniae* serotype 2 (10^7 CFU/mouse i.n.), n=10-13 mice/grp, plotted as mean +/- SEM. (B-G) Percent and total number of neutrophils (B), TNFα$^+$ neutrophils (G), Spn-FITC$^+$ neutrophils, detected following 1 hour incubation with or without FITC-labeled heat-

killed *S. pneumoniae*, (D), inflammatory monocytes (E), DCs (F), and macrophages (G) detected by flow cytometry in the middle ear of bone marrow chimeric mice from (A). Data pooled from 3 independent experiments. Box boundaries indicate the 25th and 75th percentiles, with a horizontal line representing the median and whiskers indicating minimum and maximum values. *p<.05, **p<.01, Welch's t test (A), one-way ANOVA with Sidak's post hoc test (B-G).

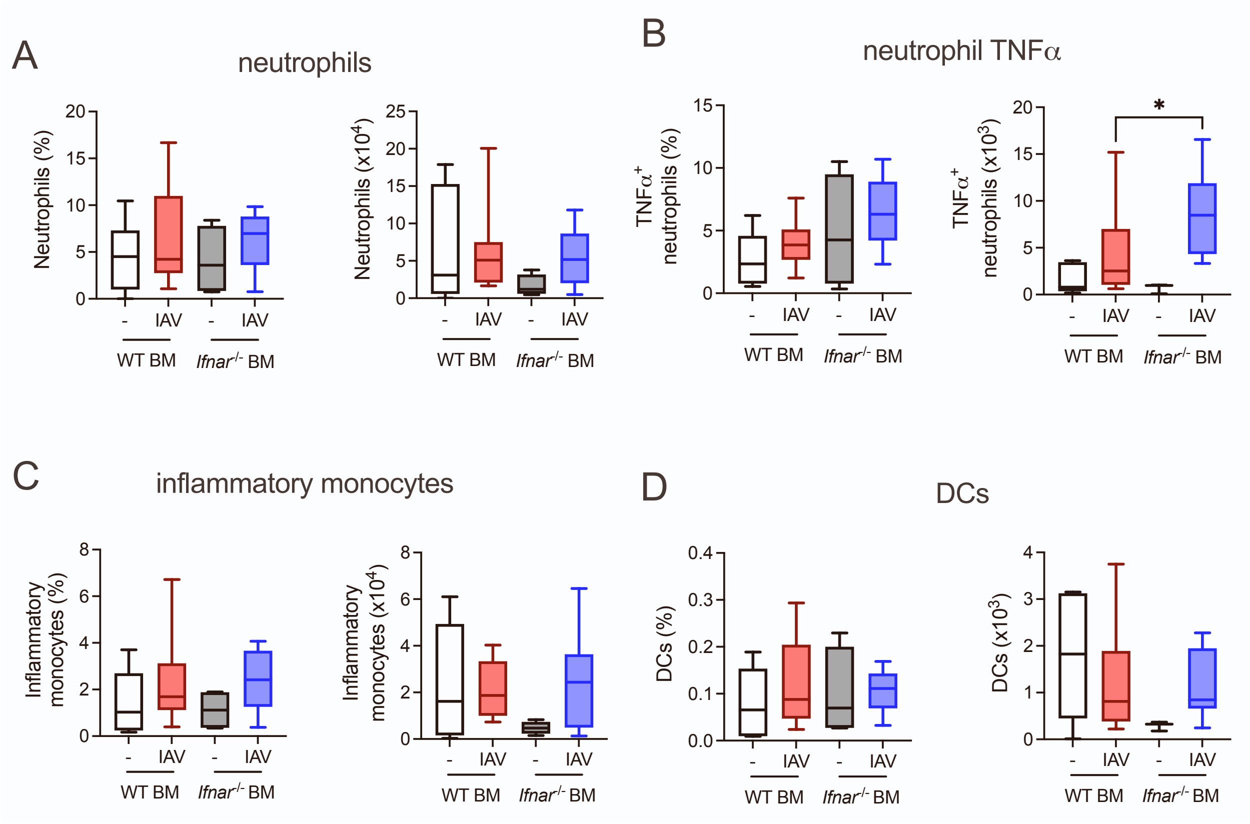

Supplementary Figure 5 (related to Figure 6). Impact of IFNAR signaling in irradiation-sensitive cells on immune cell recruitment and activation in the middle ear during *S. pneumoniae* serotype 7F infection. (A-D) Percent and total number of neutrophils (A), TNFα$^+$ neutrophils (B), inflammatory monocytes (C), and DCs (D) detected by flow cytometry in the middle ear of irradiated WT recipients of WT or *Ifnar*$^{-/-}$ bone marrow with or without IAV co-infection (10^5 PFU/mouse i.n.) 72 hours prior to challenge with *S. pneumoniae* serotype 7F (10^5 CFU/mouse i.n.), n=7-10 mice/grp. Data pooled from 3 independent experiments. Box boundaries indicate the 25th and 75th percentiles, with a horizontal line representing the median and whiskers indicating minimum and maximum values. *p<.05, one-way ANOVA with Sidak's post hoc test (B-G).

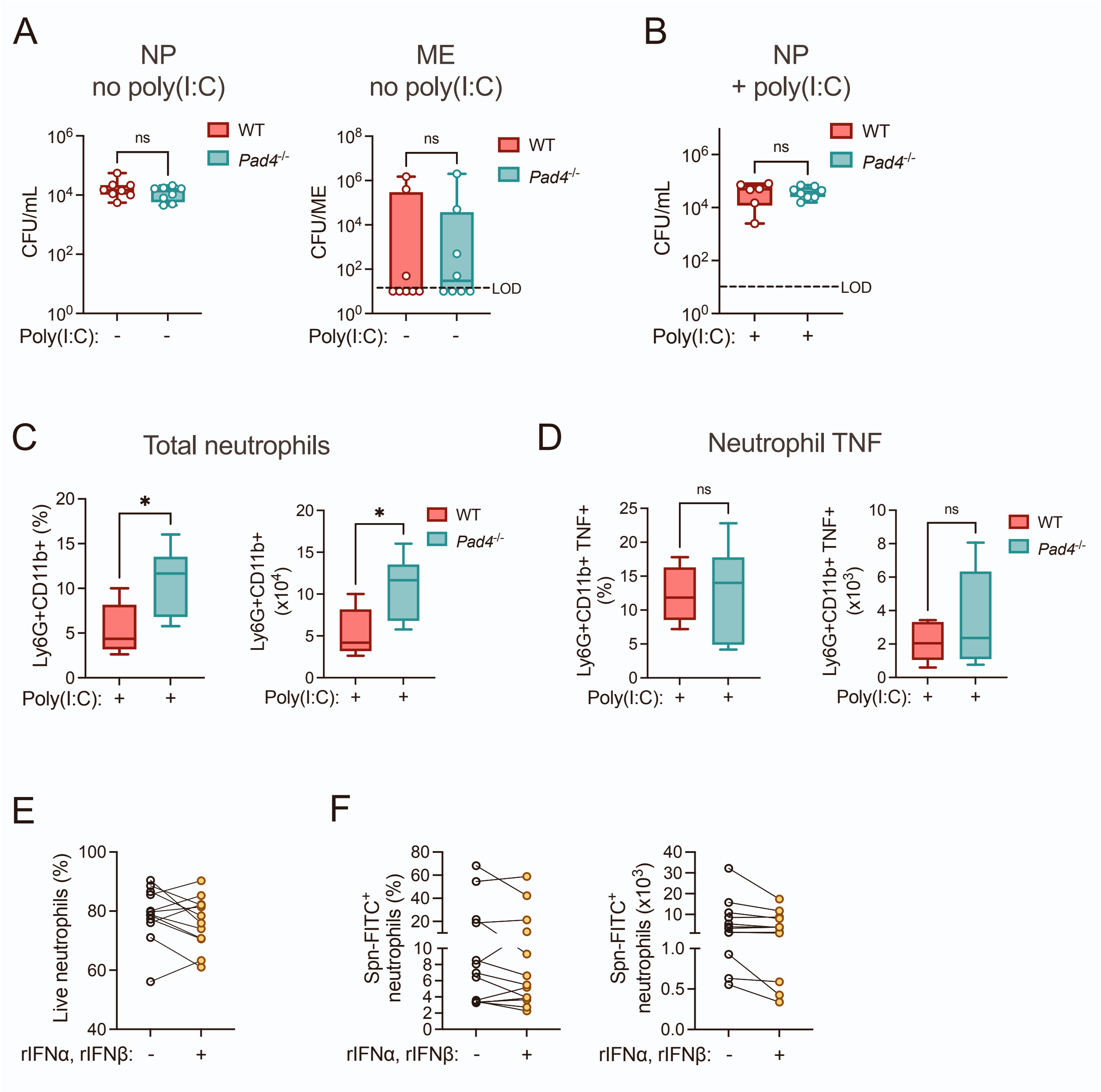

Supplementary Figure 6 (related to Figure 7). Impact of PAD4 deficiency and type I IFN exposure on *S. pneumoniae* infection and neutrophil activation. (A) Burden of *S. pneumoniae* serotype 7F detected in the nasopharynx (NP) and middle ear (ME) in WT or *Pad4*$^{-/-}$ mice at 24 hours post-infection (10^5 CFU/mouse i.n.). (B) Burden of *S. pneumoniae* detected in the nasopharynx of WT or *Pad4*$^{-/-}$ mice at 24 hours post-infection with co-treatment with poly(I:C) (50 µg/mouse i.n.). (C-D) Percent and total number of neutrophils (C) and TNFα$^+$ neutrophils (D) detected by flow cytometry in the middle ear of WT or *Pad4*$^{-/-}$ mice at 24 hours post-infection with *S. pneumoniae* and co-treatment with poly(I:C). (E) Percent of live neutrophils detected by flow cytometry with live/dead staining for neutrophils purified from human blood with or without 1 hour exposure to IFNα2 and IFNβ (1 µg/sample), n=12 subjects. (F) Percent and total number of Spn-FITC$^+$ neutrophils, detected following 1 hour incubation with or without FITC-labeled heat-killed *S. pneumoniae*, for neutrophils purified from human blood with or without 3 hour exposure to IFNα2 and IFNβ (1 µg/sample), n=13 subjects. Data pooled from 3 (A-D) or 5 (E-F) independent experiments. Box boundaries indicate the 25th and 75th percentiles, with a horizontal line representing the median and whiskers indicating minimum and maximum values. LOD = limit of detection. *p<.05, Mann-Whitney U test.
