# Supplementary material for: Type I interferon signaling in hematopoietic cells impairs neutrophil antibacterial function in the middle ear during viral co-infection
Source: Cell Rep Med. 2026 Jun 3;7(6):102846. doi: 10.1016/j.xcrm.2026.102846 (PMC13293972; doi:10.1016/j.xcrm.2026.102846)
Supplement: Document S2. Article plus supplemental information [file mmc2.pdf]

Cell Reports Medicine

Article

Type I interferon signaling in hematopoietic cells impairs neutrophil antibacterial function in the middle ear during viral co-infection

Graphical abstract

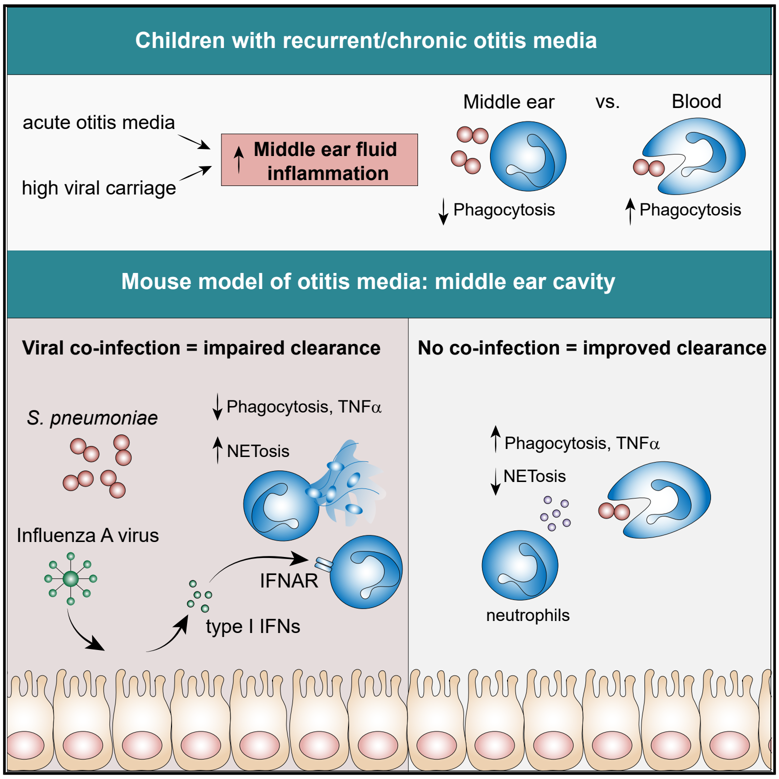

Highlights

- Virus-induced type I interferon increases bacterial infection of the middle ear
- The cellular targets of type I interferon differ for lung versus ear co-infections
- Type I interferon reduces the phagocytic capacity of middle ear neutrophils
- Middle ear NETs induced by type I IFN impair clearance of *S. pneumoniae*

Authors

Steven C. Shaw, Taylor L. Jamil, Gabriela Heslop, ..., J. Kirk Harris, Sarah A. Gitomer, Sarah E. Clark

Correspondence

sarah.e.clark@cuanschutz.edu

In brief

Shaw et al. investigate how immune modulation by viruses worsens bacterial infection in the middle ear, the most common site of childhood infection. In a pathway distinct from lung co-infections, virus induction of a type I interferon response targets myeloid cells, impairing middle ear neutrophil antibacterial function.

Shaw et al., 2026, Cell Reports Medicine 7, 102846
June 16, 2026 © 2026 The Author(s). Published by Elsevier Inc.
https://doi.org/10.1016/j.xcrm.2026.102846

Cell Reports Medicine

CellPress
OPEN ACCESS

Article

Type I interferon signaling in hematopoietic cells impairs neutrophil antibacterial function in the middle ear during viral co-infection

Steven C. Shaw,[1] Taylor L. Jamil,[1] Gabriela Heslop,[1] Jeremy T. Fleck,[1] Wyatt Johnson,[1] Brian P. Lorenz,[1] Zoe Drigot,[1] J. Kirk Harris,[2] Sarah A. Gitomer,[1,3] and Sarah E. Clark[1,4,*]
[1]Department of Otolaryngology – Head & Neck Surgery, University of Colorado School of Medicine, Aurora, CO 80045, USA
[2]Department of Pediatrics, University of Colorado School of Medicine, Aurora, CO 80045, USA
[3]Children's Hospital Colorado, Department of Otolaryngology - Head & Neck Surgery, University of Colorado School of Medicine, Aurora, CO 80045, USA
[4]Lead contact
*Correspondence: sarah.e.clark@cuanschutz.edu
https://doi.org/10.1016/j.xcrm.2026.102846

SUMMARY

The most common infection of childhood is otitis media, caused by bacterial infection of the middle ear. In children with otitis media, middle ear inflammation corresponds with acute infection and greater viral pathogen carriage. In mice, induction of a type I interferon (IFN) response is sufficient to increase *Streptococcus pneumoniae* middle ear infection. In contrast to the mechanisms of virus-induced immune dysfunction described in the lungs, the critical cellular targets of type I IFN are irradiation-sensitive cells, namely, myeloid cells. Type I IFN receptor (IFNAR) signaling impairs neutrophil phagocytic capacity, corresponding with reduced *S. pneumoniae* clearance from the middle ear. Middle ear neutrophils from children with otitis media also demonstrate impaired phagocytosis. Last, type I IFN-driven neutrophil extracellular traps (NETs) reduce the number of functional neutrophils in mouse and human samples. These findings highlight neutrophil function as a key target of virus-associated immune dysregulation during otitis media.

INTRODUCTION

The immune response to viral infection can predispose to secondary bacterial infection. While the mechanisms of viral impairment of antibacterial immunity in the lungs are well-defined, it is not clear how viral infection alters antibacterial defense in the middle ear, the most common site of infection during childhood. Approximately 75% of children experience at least one episode of middle ear infection, or otitis media (OM), by age three, and ∼30% of these experience six or more episodes by age seven.[1,2] Persistent OM with effusion can lead to permanent hearing loss or less frequently, life-threatening meningitis.[3,4] OM is the most common diagnosis for antibiotic prescription in children under six years old, and the leading indication for pediatric visits, hospitalization, and surgery in this age group.[5,6] *Streptococcus pneumoniae* (the pneumococcus) and non-typeable *Haemophilus influenzae* (NTHi) are the two most common causes of OM, followed by *Moraxella catarrhalis*.[7,8]

Middle ear infection occurs following bacterial ascension from the site of colonization in the nasopharynx through the Eustachian tube.[3] One of the most important risk factors for OM, aside from colonization with a bacterial otitis pathogen, is viral infection.[9] Several reports indicate acute OM as a complication of viral upper respiratory tract infection, with primary viral infections in the nasopharynx associated with bacterial OM 3–4 days later.[10–12] Viral co-infections are common, representing up to two-thirds of all OM cases.[13,14] Among the viruses associated with bacterial OM, rhinovirus, influenza A virus (IAV), and respiratory syncytial virus are the most prevalent.[15] The importance of viral infection in driving OM is reflected in the experimental approaches to study OM, as bacterial middle ear infection is frequently induced by intranasal co-infection with IAV to facilitate bacterial invasion of the middle ear.[16–19] Despite the strong association between viral infection and bacterial OM, the impact of viral co-infection on immune defense against bacterial infection in the middle ear is poorly understood.

In the lungs, viral infections interfere with antibacterial immune defense. Viral induction of a type I interferon (IFN) response is important for this process, based on loss of virus-mediated enhancement of *S. pneumoniae* infection in type I IFN receptor (IFNAR)-deficient mice.[20–22] In the lungs, IFNAR signaling restricts neutrophil recruitment and production of myeloperoxidase (MPO).[20–22] While neutrophil phagocytosis generally correlates with bacterial killing, neutrophil extracellular traps (NETs) are not bactericidal for *S. pneumoniae* and were not associated with *S. pneumoniae* killing during IAV co-infection in the lungs.[23,24] NETs are the dominant host immune signature detected in the middle ear of patients with OM by proteomic analysis and occur in animal models of OM.[25,26] The impact of type I IFN signaling on the balance between protective and

 © 2026 The Author(s). Published by Elsevier Inc.
This is an open access article under the CC BY license (http://creativecommons.org/licenses/by/4.0/).

non-protective neutrophil functions during viral co-infections in the middle ear is unknown.

Here, we use a mouse model recapitulating the natural route of middle ear infection to interrogate the impact of virus-induced type I IFN on antibacterial defense during acute OM. Our data indicate that induction of type I IFN is both necessary and sufficient to enhance middle ear infection. In contrast to the lungs, IAV co-infection enhancement of *S. pneumoniae* infection in the middle ear is shown to be dependent on irradiation-sensitive myeloid cells, with type I IFN-mediated neutrophil dysfunction serving as the central component of defective bacterial clearance.

RESULTS

Viral carriage and acute infection correlate with middle ear inflammation in children with otitis media

Carriage of bacterial OM pathogens and respiratory tract viral pathogens was measured in children with OM diagnosed as recurrent acute OM (rAOM) or chronic OM with effusion (COME). Swabs were collected from the nasal cavity and nasopharynx. In children undergoing ear tube surgery (myringotomy), fluid was collected from the middle ear cavity. As expected, the genera containing the top three OM bacterial pathogens, *Haemophilus*, *Moraxella*, and *Streptococcus*, were the most abundant operational taxonomic units detected in the nasal cavity and nasopharynx by 16S rRNA sequencing across subjects, except for two children with high abundance of *Staphylococcus* (Figure 1A). Similar dominance of the genera representing bacterial OM pathogens was apparent in middle ear samples. Swabs were also analyzed for viral pathogen carriage using a clinical respiratory pathogen panel. Carriage of viral pathogens was particularly high in this population, as viral pathogens were detected in ~83% (20 of 24) of subjects (Figure 1B). Among these, ~40% had three or more viral pathogens detected. Notably, total carriage of viral pathogens in this cohort was over 50% higher than that reported for asymptomatic children, and multiple viral pathogens were only detected in 10%–13% of children without OM diagnosis.[27,28]

We next profiled middle ear fluid collected from children undergoing ear tube surgery for rAOM or COME for cytokine and chemokine levels by multiplex analysis. The concentrations of pro-inflammatory cytokines and chemokines including CXCL2, TGFβ, IFNγ, IL-1β, MCP-1, IL-6, IL-10, IP-10 (CXCL10), and IL-8 were significantly higher in children with a clinical diagnosis of acute OM (active infection), compared to those without active infection (Figure 1C; Figure S1A). Samples collected from children with active infection also had higher rates of purulent, or "pus-like," effusions, which were noted for samples from 58% of children with acute OM compared to 0% in those without active infection (Figures S1B and 1C). This cohort was demographically similar, with comparable influenza and pneumococcal vaccine coverage (Table 1). Based on the observation that many children in this cohort carried one or more viral pathogens in their nasal cavity or nasopharynx, we also compared cytokine/chemokine levels in subjects with low (0–2) versus high (3 or more) number of viral pathogens detected. The concentrations of TNFα, IL-4, IL-2, IL-12p70, IL-1β, IL-6, IL-10, and IP-10 (CXCL10) were significantly higher in children with a high number (3+) of viral pathogens detected, with trending increases in several other pro-inflammatory cytokines and chemokines also elevated during acute OM (Figure 1D; Figure S1D). IP-10 (CXCL10) is an IFN-induced chemokine directly stimulated by type I IFN through an IFN-stimulated response element in the promoter[29] and was significantly elevated in subjects infected with influenza followed by *S. pneumoniae* in a human challenge study.[30] Type I IFNs themselves are rapidly produced and degraded, with extremely low detection reported in clinical samples necessitating reliance on IFN-induced responses, including IP-10 (CXCL10).[31,32] Consistent with this, while minimal IFNα2 and IFNβ was detected in this cohort (Figure S1E), IP-10 was significantly elevated among children with both active infection and a higher number of viral pathogens. These findings highlight correlations between active infection and viral pathogen carriage with middle ear inflammation in children with OM.

Type I IFN induction is sufficient to enhance bacterial infection of the middle ear

We next used a murine infection model to interrogate how viral co-infection impacts the immune response during acute OM. Mice were intranasally infected with IAV strain x31 three days prior to intranasal challenge with *S. pneumoniae* serotype 7F, with *S. pneumoniae* burdens in the middle ear measured 24 h post-infection (Figure 2A). Compared to mice infected with *S. pneumoniae* alone, burdens in both the nasopharynx and middle ear were increased by IAV co-infection in a dose-dependent manner (Figure 2A).

To separate the effects of viral induction of a type I IFN response from other consequences of active viral replication, we tested the effect of co-inoculation with the viral analog poly(I:C), a TLR3 agonist that induces type I IFN. The impact of poly(I:C) on *S. pneumoniae* middle ear infection was tested across three serotypes with different levels of baseline middle ear invasion rates of 0% (serotype 2), 53% (serotype 7F), and 86% (serotype 19F) (Figures 2B–2D). For all three serotypes, intranasal co-treatment with poly(I:C) significantly increased *S. pneumoniae* burdens in the middle ear at 24 h post-infection (Figures 2B–2D). For serotype 2, the percentage of mice with detectable middle ear infection rose from 0% to 62% with poly(I:C) treatment, and for serotype 7F, all mice co-treated with poly(I:C) had detectable infection (100%) compared to 53% in mice infected with *S. pneumoniae* alone (Figures 2B and 2C). While baseline infection was highest for serotype 19F, poly(I:C) treatment increased median *S. pneumoniae* burdens in the middle ear from ~10^3 to over 10^5 colony-forming units (CFUs) (Figure 2D). In each case, poly(I:C) had no impact on nasopharyngeal burdens of *S. pneumoniae*, indicating that elevated middle ear infections were not a direct consequence of more bacteria in the nasopharynx (Figures 2B–2D). Of the three serotypes we assessed, serotype 7F and 19F are strongly associated with OM, leading to their inclusion in the pneumococcal conjugate vaccines, despite which serotype 19F continues to be identified in OM cases.[33,34] Serotype 2 was originally associated with meningitis, is included in the non-conjugate pneumococcal vaccines, and is an important

CellPress
OPEN ACCESS

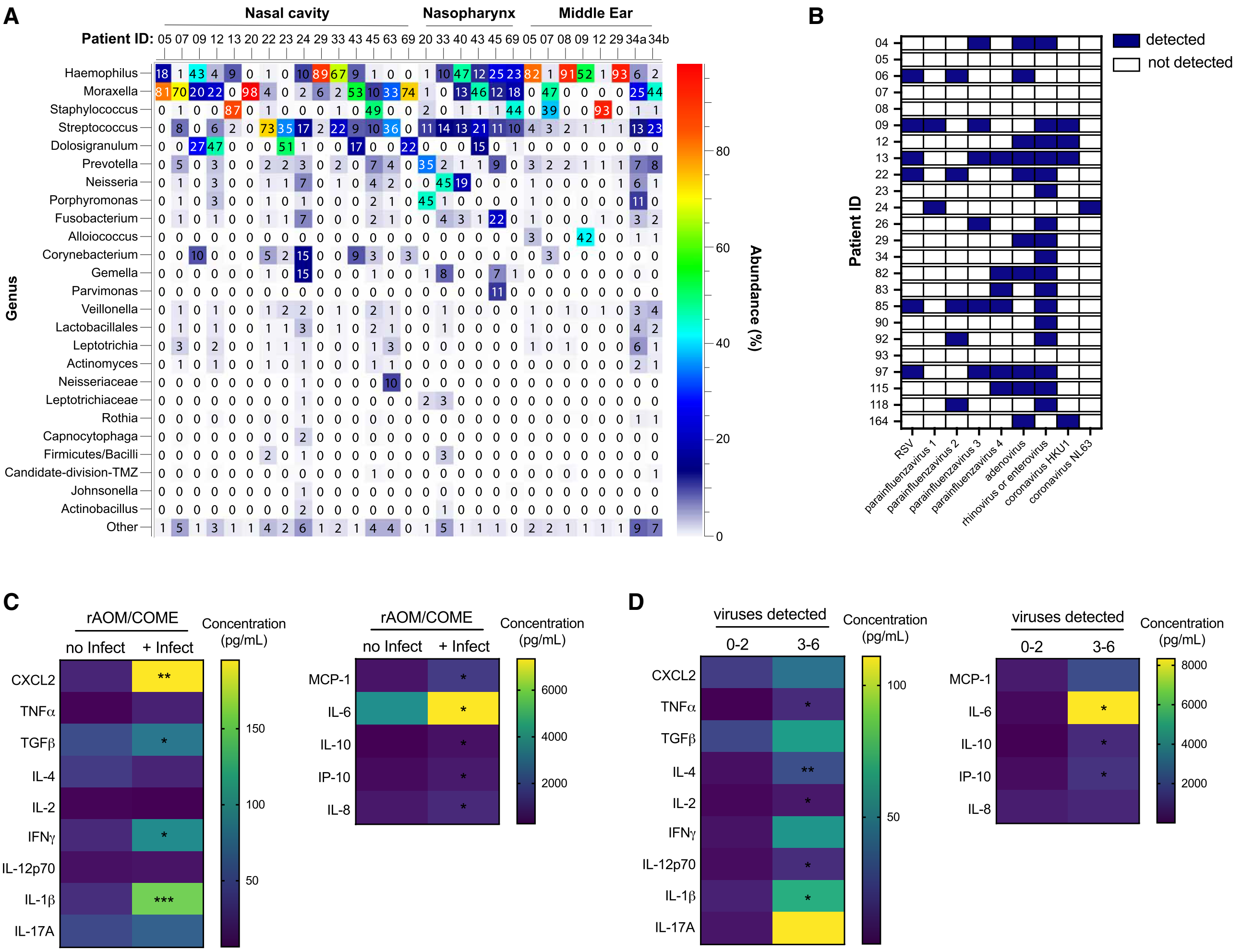

Figure 1. Viral carriage and acute infection correlate with middle ear inflammation in children with otitis media

(A) Percent abundance of bacterial genera in samples collected from the nasal cavity, nasopharynx, or middle ear of children with recurrent acute OM (rAOM) or chronic OM with effusion (COME).

(B) Viral pathogens detected in nasal cavity or nasopharyngeal swabs collected from children with rAOM or COME, with filled boxes indicating viruses detected for each patient.

(C) Concentration of cytokines/chemokines detected in middle ear fluid samples from children with rAOM or COME with a clinical diagnosis of acute OM (+Infect, $n = 18$ subjects) or no diagnosis of acute OM (no Infect, $n = 31$ subjects). Median concentrations displayed.

(D) Concentration of cytokines/chemokines detected in middle ear fluid samples from children with rAOM or COME with 0–2 individual viruses detected ($n = 8$ subjects) or 3–6 viruses detected ($n = 8$ subjects). Samples for (D) included all middle ear fluid collections from subjects tested for viruses. Viral testing was completed based on swab availability. Median concentrations displayed. Samples at or below the limit of detection are reported as 0 pg/mL.

*$p < 0.05$, **$p < 0.01$, ***$p < 0.001$, Mann-Whitney U test.

benchmark strain in the pneumococcal field.[35] Together, these data indicate a conserved effect for poly(I:C), which was sufficient to enhance middle ear *S. pneumoniae* burdens and infection rate.

IFNAR signaling is required for type I IFN-enhanced bacterial infection in the middle ear

To confirm induction of type I IFN by poly(I:C) treatment, IFNβ was measured in middle ear homogenates at 24 h post-infection in mice infected with *S. pneumoniae* serotype 7F with or without poly(I:C) co-inoculation. The level of IFNβ in the middle ear of mice treated with poly(I:C) was significantly higher than that of mice infected with *S. pneumoniae* alone, in which there was a slight but non-significant amount of IFNβ detected compared to naive mice (Figure 3A). To assess the impact of type I IFN alone, mice were co-inoculated with recombinant IFNα2 and IFNβ in place of poly(I:C). Treatment with rIFNα2 and rIFNβ significantly increased *S. pneumoniae* burdens in the middle ear, compared to untreated mice (Figure 3B). As with poly(I:C), nasopharyngeal burdens were unaffected. These data indicate that type I IFNs are sufficient to increase *S. pneumoniae* middle ear infection.

To investigate whether type I IFN signaling is required for poly(I:C)-enhanced *S. pneumoniae* infection, mice were treated

Table 1. Patient sample information

Characteristic	With AOM at time of surgery (n = 18)	No AOM at time of surgery (n = 34)
Age in years, median (range)	2 (0.67–8)	3 (1–10)
Sex, %		
Male	66.7	52.9
Female	33.3	47.1
Race, %		
White	94.4	91.2
Black/African American	5.6	2.9
Other	0	5.9
Ethnicity, %		
Not Hispanic or Latino	77.8	82.4
Hispanic or Latino	22.2	17.6
Vaccination, %		
Influenza	61.1	73.5
COVID-19	62.5	50.0
Pneumococcal conjugate	83.3	88.2

AOM, acute otitis media.

with anti-IFNAR antibody to block IFNAR signaling. In mice co-inoculated with poly(I:C), *S. pneumoniae* serotype 7F burdens in the middle ear were significantly lower when treated with anti-IFNAR antibody compared to isotype-treated controls (Figure 3C). The importance of IFNAR signaling was next assessed in IFNAR-deficient mice. While poly(I:C) significantly increased *S. pneumoniae* middle ear burdens in wild-type (WT) mice, middle ear burdens in IFNAR-deficient mice treated with poly(I:C) were similar to those in WT mice without poly(I:C) treatment (Figure 3D). *S. pneumoniae* middle ear burdens were also significantly lower in IFNAR-deficient mice treated with poly(I:C) compared to WT mice treated with poly(I:C). In IFNAR-deficient mice, burdens were similar regardless of poly(I:C) treatment (Figure S2A). Neither anti-IFNAR antibody treatment nor IFNAR deficiency impacted *S. pneumoniae* burdens in the nasopharynx, which were unaltered by poly(I:C) (Figures 3C and 3D). Together these findings indicate that IFNAR signaling is required for poly(I:C)-enhanced *S. pneumoniae* infection of the middle ear.

Type I IFN-mediated enhancement of middle ear infection is not dependent on induction of IFNγ or suppression of neutrophil recruitment

In addition to type I IFN, type II IFN (IFNγ) has been described as an important mediator of virus-induced hypersensitivity to *S. pneumoniae* infections in the lungs. To investigate the impact of poly(I:C) treatment on the overall cytokine response, including IFNγ production, middle ear fluid was collected from mice infected with *S. pneumoniae* serotype 7F with or without poly(I:C) for multiplex analysis at 24 h post-infection. Overall, *S. pneumoniae* infection was characterized by a significant increase in the chemokine IP-10 (CXCL10) compared to naive mice, with significantly higher IP-10 in mice treated with poly(I:C) (Figure 4A). Mice infected with *S. pneumoniae* and co-inoculated with poly(I:C) also had significantly elevated levels of TNFα in middle ear fluid compared to mice infected with *S. pneumoniae* alone and naive mice. The cytokines IL-6 and IL-10 were significantly higher in mice infected with *S. pneumoniae* and treated with poly(I:C) compared to naive mice, with no statistical difference from levels detected in mice infected with *S. pneumoniae* alone or for *S. pneumoniae* alone versus naive for IL-6. Middle ear fluid levels of IFNγ were low (under 50 pg/mL), with no differences between groups.

Considering the critical role described for IFNγ as a mechanism of viral enhancement of bacterial infection in the lungs, we examined the effect of blocking this response with anti-IFNγ antibody treatment. Blockade of IFNγ had no impact on poly(I:C) enhancement of *S. pneumoniae* middle ear infection, as *S. pneumoniae* burdens were significantly higher in poly(I:C)-treated mice compared to untreated mice regardless of anti-IFNγ antibody exposure (Figure 4B). Serum levels of IFNγ were significantly lower at 24 h post-*S. pneumoniae* middle ear infection with poly(I:C) treatment compared to levels detected in serum from mice 24 h post-*S. pneumoniae* lung infection, and anti-IFNγ antibody treatment abrogated this response (Figure S2B). These data suggest that poly(I:C) enhancement of *S. pneumoniae* middle ear infection is IFNγ independent.

We next sought to understand whether the type I IFN-induced increase in *S. pneumoniae* middle ear burdens was the result of altered neutrophil recruitment, as has been described for IAV co-infection in the lungs.[20,21] *S. pneumoniae* burdens, neutrophil-recruiting chemokines, and neutrophil infiltration to the middle ear were analyzed over a time course in mice treated with or without poly(I:C). *S. pneumoniae* serotype 7F middle ear burdens increased over time, with 50% of mice infected in the middle ear by 6 h post-infection, rising to ~60% of mice with detectable infection at 12 and 24 h post-infection without poly(I:C) treatment (Figure S2C). Middle ear infection rates in mice co-inoculated with poly(I:C) were similar at 6 and 12 h post-infection but were significantly elevated by 24 h post-infection, at which point the percentage of infected mice increased from ~60% in mice infected with *S. pneumoniae* alone to 100% in poly(I:C)-treated mice. *S. pneumoniae* nasopharyngeal burdens remained relatively stable, with similar levels detected regardless of poly(I:C) treatment (Figure S2C). At 24 h post-infection, the neutrophil-recruiting chemokines CXCL1 and CXCL2 were significantly higher in mice co-inoculated with poly(I:C) compared to mice infected with *S. pneumoniae* alone (Figure 4C), suggesting an elevated neutrophil chemokine response. Neutrophil recruitment to the middle ear over time was assessed by flow cytometry (Figure S2D). Despite the elevation in neutrophil-recruiting chemokines, there was no difference in the percentage or total number of neutrophils recruited to the middle ear in mice infected with *S. pneumoniae* with or without poly(I:C) treatment (Figure 4D). To assess the impact of IFNAR signaling on neutrophil recruitment to the middle ear during *S. pneumoniae* infection, neutrophils were also quantified in WT versus IFNAR-deficient mice co-inoculated with poly(I:C). IFNAR deficiency had

CellPress
OPEN ACCESS

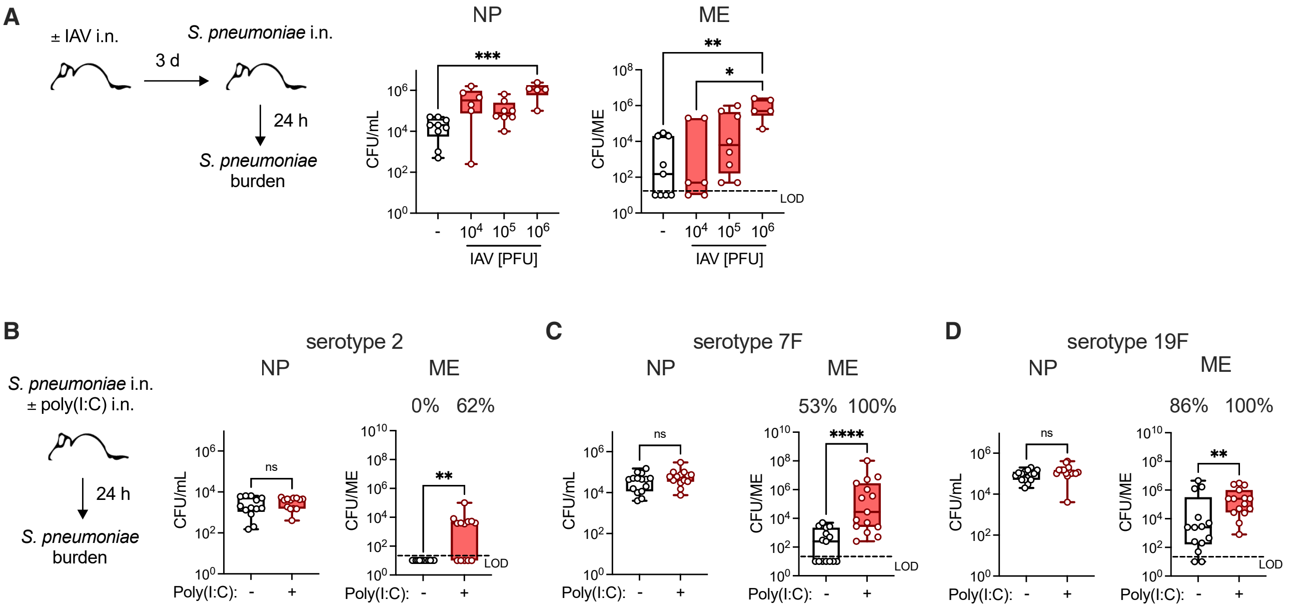

Figure 2. Type I IFN induction is sufficient to enhance bacterial infection of the middle ear

(A) Burdens of *S. pneumoniae* serotype 7F detected in the nasopharynx (NP) or middle ear (ME) of WT mice 24 h post-infection (10^5 CFU/mouse i.n. [intranasal]) with or without IAV infection 3 days prior to bacterial challenge at the indicated dose i.n. (n = 5–9 mice/grp); grp, group.

(B–D) Burdens of *S. pneumoniae* serotype 2 (10^7 CFU/mouse, n = 13 mice/grp) (B), serotype 7F (10^5 CFU/mouse, n = 15–16 mice/grp) (C), or serotype 19F (10^6 CFU/mouse, n = 15 mice/grp) (D) with or without co-treatment with poly(I:C) (50 μg/mouse i.n.) detected in the NP or ME of WT mice at 24 h post-infection. Percentages indicate mice with detectable infection in the ME.

Data pooled from 3 independent experiments. Box boundaries indicate the 25th and 75th percentiles, with a horizontal line representing the median and whiskers indicating minimum and maximum values. LOD, limit of detection. *$p < 0.05$, **$p < 0.01$, ***$p < 0.001$, ****$p < 0.0001$; Kruskal-Wallis with Dunn's post hoc test (A) and Mann-Whitney U test (B–D).

no impact on neutrophil recruitment, with similar numbers of neutrophils detected as in WT mice with or without poly(I:C) (Figure 4E). Together, these findings suggest that type I IFN-mediated enhancement of *S. pneumoniae* infection in the middle ear is not due to altered neutrophil recruitment.

IFNAR signaling attenuates middle ear neutrophil TNFα and impairs neutrophil phagocytosis of *S. pneumoniae*

We next investigated whether poly(I:C) treatment altered neutrophil activation and antimicrobial function. Neutrophil expression of the pro-inflammatory cytokine TNFα was assessed as a marker of activation by intracellular flow cytometry. For this analysis, neutrophil activation was compared in WT mice with or without poly(I:C) treatment and in IFNAR-deficient mice treated with poly(I:C) to determine the impact of IFNAR signaling. The percentage and number of neutrophils expressing TNFα were significantly reduced in WT mice treated with poly(I:C), compared to WT mice infected with *S. pneumoniae* serotype 7F alone (Figure 5A). However, TNFα expression levels were restored in IFNAR-deficient mice treated with poly(I:C), where the percentage and number of neutrophils expressing TNFα were significantly higher than in WT mice treated with poly(I:C) (Figure 5A). These findings indicate that the poly(I:C) associated impairment of neutrophil TNFα expression in the middle ear is IFNAR dependent.

An important role for neutrophils mediating middle ear bacterial defense was confirmed by significantly elevated burdens of *S. pneumoniae* following partial neutrophil depletion using anti-Ly6G antibody treatment, compared to isotype-treated controls (Figures S3A and S3B). The reduction in neutrophil TNFα following poly(I:C) treatment suggested a neutrophil activation defect associated with the impaired bacterial clearance from the middle ear. This relationship was apparent when comparing the number of TNFα+ neutrophils in mice with versus without detectable *S. pneumoniae* infection. In mice without detectable infection, indicating potential bacterial clearance, the number of TNFα+ neutrophils was significantly higher than in mice with *S. pneumoniae* infection detected (Figure 5B).

To further analyze neutrophil activation, we assessed the phagocytic capacity of neutrophils in the middle ear based on detection of fluorescein isothiocyanate (FITC)-labeled heat-killed *S. pneumoniae* uptake by flow cytometry (Figure S3C). This method detects both intracellular bacteria and a low number of tightly bound extracellular *S. pneumoniae*,[36] with ~90% of the detected signal attributed to intracellular *S. pneumoniae* (Figure S3D). Phagocytosis of *S. pneumoniae* by middle ear neutrophils was significantly reduced in mice treated with poly(I:C), compared to those infected with *S. pneumoniae* alone (Figure 5C). In this experiment, the number of neutrophils expressing TNFα was also lower in poly(I:C)-treated mice. Across all samples, the percentage of neutrophils that phagocytosed the FITC-labeled *S. pneumoniae* significantly correlated with the percentage of neutrophils expressing TNFα (Figure 5D). Intracellular production of reactive oxygen species, which contributes to bacterial

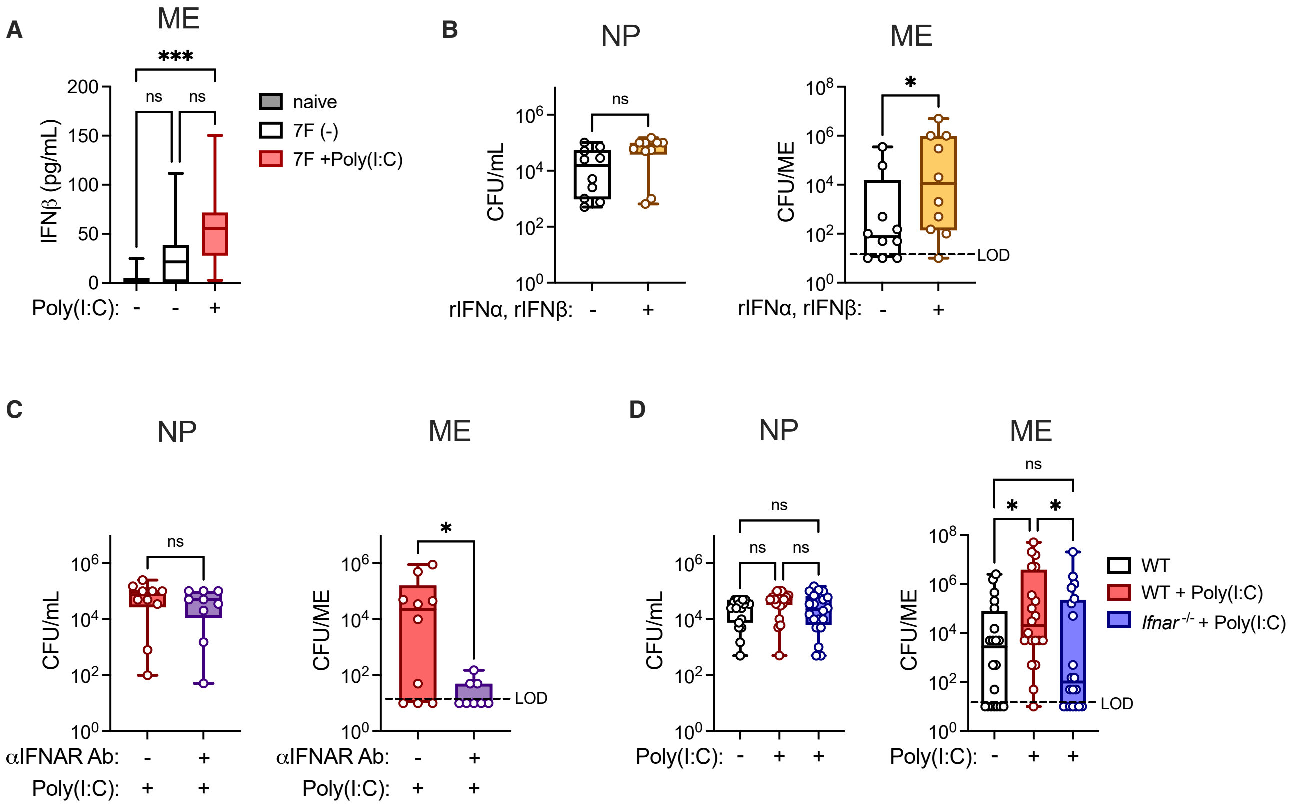

Figure 3. IFNAR signaling is required for type I IFN-enhanced bacterial infection in the middle ear

(A) Concentration of IFNβ detected in the middle ear (ME) of naive WT mice or 24 h post-infection with *S. pneumoniae* serotype 7F (10^5 CFU/mouse i.n.) with or without co-treatment with poly(I:C) (50 μg/mouse i.n.), n = 8–12 mice/grp.

(B) Burden of *S. pneumoniae* detected in WT mice at 24 h post-infection with or without co-treatment with recombinant IFNα2 and IFNβ (1 μg each/mouse i.n.), n = 10 mice/grp.

(C) Burden of *S. pneumoniae* detected in WT mice at 24 h post-infection with or without co-treatment with anti-IFNAR antibody or isotype control antibody (200 μg/mouse i.p.), n = 9–10 mice/grp.

(D) Burden of *S. pneumoniae* detected in WT or *Ifnar*$^{-/-}$ mice at 24 h post-infection with or without poly(I:C) (50 μg/mouse i.n.), n = 21 mice/grp.

Data pooled from 3 (A–C) or 4 (D) independent experiments. Box boundaries indicate the 25th and 75th percentiles, with a horizontal line representing the median and whiskers indicating minimum and maximum values. LOD, limit of detection. *p < 0.05, ***p < 0.001; Kruskal-Wallis with Dunn's post hoc test (A and D) and Mann-Whitney U test (B and C).

killing, was unaffected by poly(I:C) (Figure S3E). Together, these results indicate that neutrophils recruited to the middle ear following type I IFN induction by poly(I:C) are functionally impaired, with a reduced capacity to phagocytose *S. pneumoniae*.

To assess neutrophil functional capacity in children with OM, cells were collected from the blood and middle ear fluid of subjects undergoing ear tube surgery for analysis of neutrophil phagocytosis by flow cytometry (Figure S3F). While neutrophils were readily detected in both the blood and middle ear fluid in children with OM, *S. pneumoniae* phagocytosis was significantly higher for neutrophils from the blood compared to neutrophils from the middle ear (Figures 5E–5I). This relationship was maintained across the small number of patients for which we obtained paired samples of blood and middle ear fluid, where *S. pneumoniae* phagocytosis was significantly lower in the neutrophils from the middle ear (Figure 5I). These findings are consistent with a sustained defect in middle ear neutrophil phagocytic capacity in children with OM.

IFNAR signaling in irradiation-sensitive myeloid cells impairs neutrophil phagocytosis and pneumococcal clearance from the middle ear during viral co-infection

To delineate the contribution of IFNAR expression on hematopoietic cells, including neutrophils and other myeloid cells, to impaired *S. pneumoniae* clearance during viral co-infection, bone marrow chimeric mice were generated. Irradiated CD45.1+ WT mice received bone marrow from CD45.2+ WT or IFNAR-deficient mice, resulting in mice with normal expression of IFNAR on all epithelial cells and either WT or IFNAR-deficient hematopoietic cells. Chimeric mice were infected with IAV x31 three days prior to *S. pneumoniae* infection, and bacterial burdens were analyzed 24 h post-infection. IAV x31 co-infection caused mild disease, indicated by loss of up to 10% starting body weight, compared to negligible weight loss in mice infected with *S. pneumoniae* alone (Figure S4A), as expected. Importantly, IAV-infected recipients of WT and IFNAR-deficient bone marrow exhibited similar disease severity, facilitating analysis

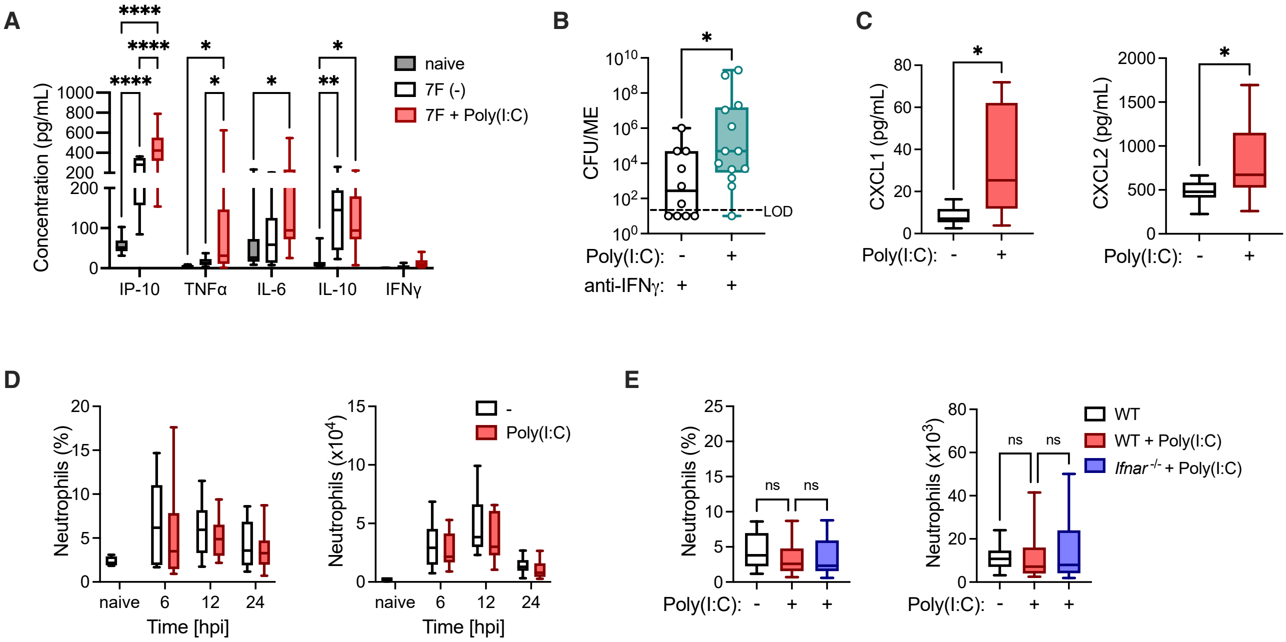

Figure 4. Type I IFN-mediated enhancement of middle ear infection is not dependent on induction of IFNγ or suppression of neutrophil recruitment

(A) Concentration of IP-10 (CXCL10), TNFα, IL-6, IL-10, and IFNγ detected in the middle ear of naive WT mice or 24 h post-infection with *S. pneumoniae* serotype 7F (10^5 CFU/mouse i.n.) with or without co-treatment with poly(I:C) (50 μg/mouse i.n.), n = 10–11 mice/grp.

(B) Burden of *S. pneumoniae* detected in the middle ear at 24 h post-infection with or without poly(I:C) in mice treated 24 h prior to infection with anti-IFNγ antibody (200 μg/mouse i.p.), n = 11–13 mice/grp.

(C) Concentration of CXCL1 and CXCL2 detected in the middle ear of WT mice 24 h post-infection with *S. pneumoniae* with or without poly(I:C), n = 10 mice/grp.

(D) Percent and total number of neutrophils detected by flow cytometry in the middle ear of WT mice at 0 (naive, n = 4 mice), 6 (n = 10 mice/grp), 12 (n = 10 mice/grp), and 24 (n = 19 mice/grp) hours post-infection with *S. pneumoniae* with or without poly(I:C).

(E) Percent and total number of neutrophils detected by cytometry in the middle ear of WT or *Ifnar*$^{-/-}$ mice at 24 h post-infection with or without poly(I:C), n = 15 mice/grp.

Data pooled from 3 independent experiments. Box boundaries indicate the 25th and 75th percentiles, with a horizontal line representing the median and whiskers indicating minimum and maximum values. LOD, limit of detection. *$p < 0.05$, **$p < 0.01$, ****$p < 0.0001$; two-way ANOVA with Tukey's post hoc test (A), Mann-Whitney U test (B and C), and Kruskal-Wallis with Dunn's post hoc test (D and E).

of bacterial control and antibacterial immune defense between these groups.

In recipients of WT bone marrow, IAV infection significantly increased *S. pneumoniae* serotype 2 burdens in both the nasopharynx and middle ear (Figure 6A). In contrast, there was no difference in *S. pneumoniae* burdens for recipients of IFNAR-deficient bone marrow regardless of IAV co-infection (Figure 6A). Outcomes were similar for chimeric mice infected with *S. pneumoniae* serotype 7F, with a trend toward elevated nasopharyngeal burdens and significantly higher middle ear burdens in recipients of WT bone marrow following IAV co-infection, but no difference in bacterial burdens for recipients of IFNAR-deficient bone marrow with or without IAV infection (Figure 6B). These data indicate that IFNAR expression on irradiation-sensitive hematopoietic cells contributes to impaired *S. pneumoniae* clearance from the middle ear during viral co-infection.

Differences in *S. pneumoniae* burdens correlated with changes in neutrophil activation and phagocytic capacity for recipients of WT versus IFNAR-deficient bone marrow in IAV co-infected mice. For *S. pneumoniae* serotype 2 infections, IAV co-infected recipients of IFNAR-deficient bone marrow had a significantly higher percentage and total number of TNFα+ neutrophils, compared to IAV co-infected recipients of WT bone marrow (Figure 6C; Figure S4C). Co-infected recipients of IFNAR-deficient bone marrow also had a recovered neutrophil phagocytic response, with significantly increased uptake of FITC-labeled *S. pneumoniae* by middle ear neutrophils compared to recipients of WT bone marrow (Figure 6D; Figure S4D). Similarly, for *S. pneumoniae* serotype 7F infections, IAV co-infected recipients of IFNAR-deficient bone marrow had a significantly increased number of TNFα+ neutrophils compared to IAV co-infected recipients of WT bone marrow (Figure 6E; Figure S5B). Together, these data indicate that IFNAR signaling in hematopoietic cells reduces middle ear neutrophil activation and *S. pneumoniae* phagocytosis during viral co-infection, correlating with increased burdens of *S. pneumoniae* in the middle ear.

As with poly(I:C) treatment, IFNAR expression had no impact on the total percentage and number of neutrophils in the middle ear during IAV co-infection (Figure S4B; Figure S5A). Analysis of other myeloid cell populations in the bone marrow chimeric mice

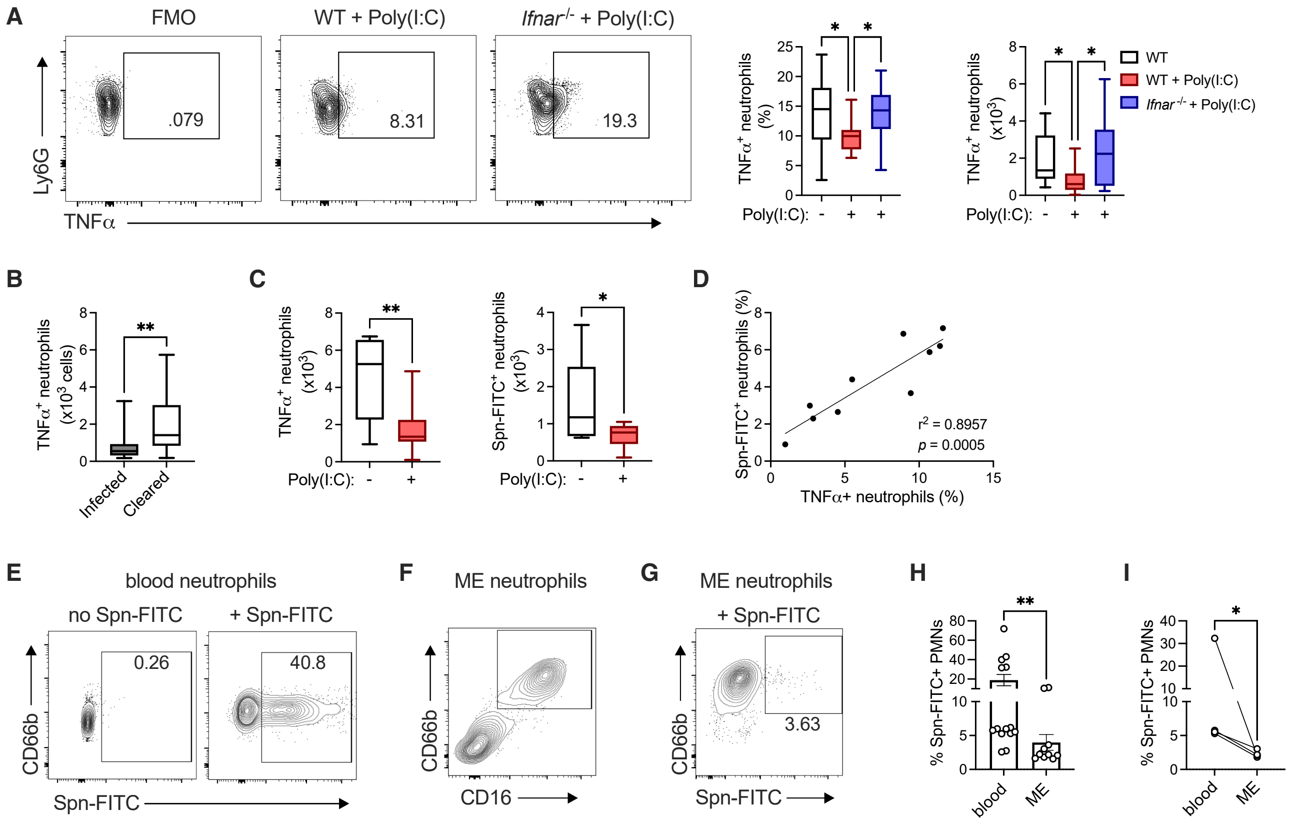

Figure 5. IFNAR signaling attenuates middle ear neutrophil TNF and impairs neutrophil phagocytosis of *S. pneumoniae*

(A) Total and percent TNFα⁺ neutrophils detected in the middle ear by intracellular flow cytometry alongside representative flow cytometry plots including fluorescence minus one (FMO) control in WT or *Ifnar*$^{-/-}$ mice at 24 h post-infection with or without co-treatment with poly(I:C) (50 μg/mouse i.n.), $n = 15$ mice/grp.

(B) Total number of TNFα$^+$ neutrophils detected in the middle ear of mice with detectable infection (Infected) or no detectable infection (Cleared) based on CFUs above the limit of detection across all mice in (A).

(C) Total number of TNFα$^+$ neutrophils and Spn-FITC$^+$ neutrophils, detected following 1 h incubation with FITC-labeled heat-killed *S. pneumoniae*, in the middle ear of WT mice at 24 h post-infection with or without poly(I:C), $n = 10$ mice/grp.

(D) Correlation between the percentage of Spn-FITC$^+$ neutrophils and TNFα$^+$ neutrophils from (C). (E) Representative flow cytometry plot of Spn-FITC$^+$ neutrophil detection for neutrophils purified from human blood.

(F and G) Representative flow cytometry plots of neutrophils (F) and Spn-FITC$^+$ neutrophils (G) for middle ear fluid samples collected from children with OM.

(H and I) Percentage of Spn-FITC$^+$ neutrophils detected in the blood versus middle ear fluid in samples collected from children with OM (H), $n = 10$–14 subjects/grp plotted as mean ± SEM, with samples from matched donors plotted in (I), $n = 4$ subjects/grp.

Data pooled from 3 independent experiments. Box boundaries indicate the 25th and 75th percentiles, with a horizontal line representing the median and whiskers indicating minimum and maximum values. *$p < 0.05$, **$p < 0.01$; Kruskal-Wallis with Dunn's post hoc test (A), unpaired *t* test (B and C), Pearson correlation coefficient (D), and Mann-Whitney U test (H and I).

confirmed a dominant role for neutrophils at 24 h post-infection with *S. pneumoniae*, as the total numbers of inflammatory monocytes, dendritic cells, and macrophages were low and accounted for a smaller percentage of total CD45$^+$ cells (Figures S4E–S4G; Figures S5C and S5D).

To extend our findings with the bone marrow chimeric mice, we evaluated the importance of IFNAR expression on myeloid cells using LysM^{cre} x IFNAR$^{fl/fl}$ mice, which have selective IFNAR deficiency on myeloid cells.[37,38] While *S. pneumoniae* serotype 2 burdens in the middle ear were significantly elevated in WT mice co-infected with IAV compared to LysM^{cre} x IFNAR$^{fl/fl}$ mice infected with *S. pneumoniae*, IAV co-infection in myeloid cell IFNAR-deficient mice failed to significantly increase *S. pneumoniae* burdens (Figure 6F). Trends were similar in the nasopharynx, with a slight but not statistically significant increase in *S. pneumoniae* burdens in WT mice co-infected with IAV compared to LysM^{cre} x IFNAR$^{fl/fl}$ mice with or without IAV co-infection (Figure 6F). Overall, these findings are consistent with an important role for IFNAR signaling on myeloid cells for impaired *S. pneumoniae* clearance from the middle ear during viral co-infection.

NETosis contributes to type I IFN impairment of *S. pneumoniae* clearance from the middle ear through the depletion of functional neutrophils

We next considered whether reduced neutrophil TNFα and phagocytosis during viral co-infection was balanced with a corresponding increase in the generation of NETs, which are ineffective for *S. pneumoniae* killing.[39] In poly(I:C)-treated mice infected

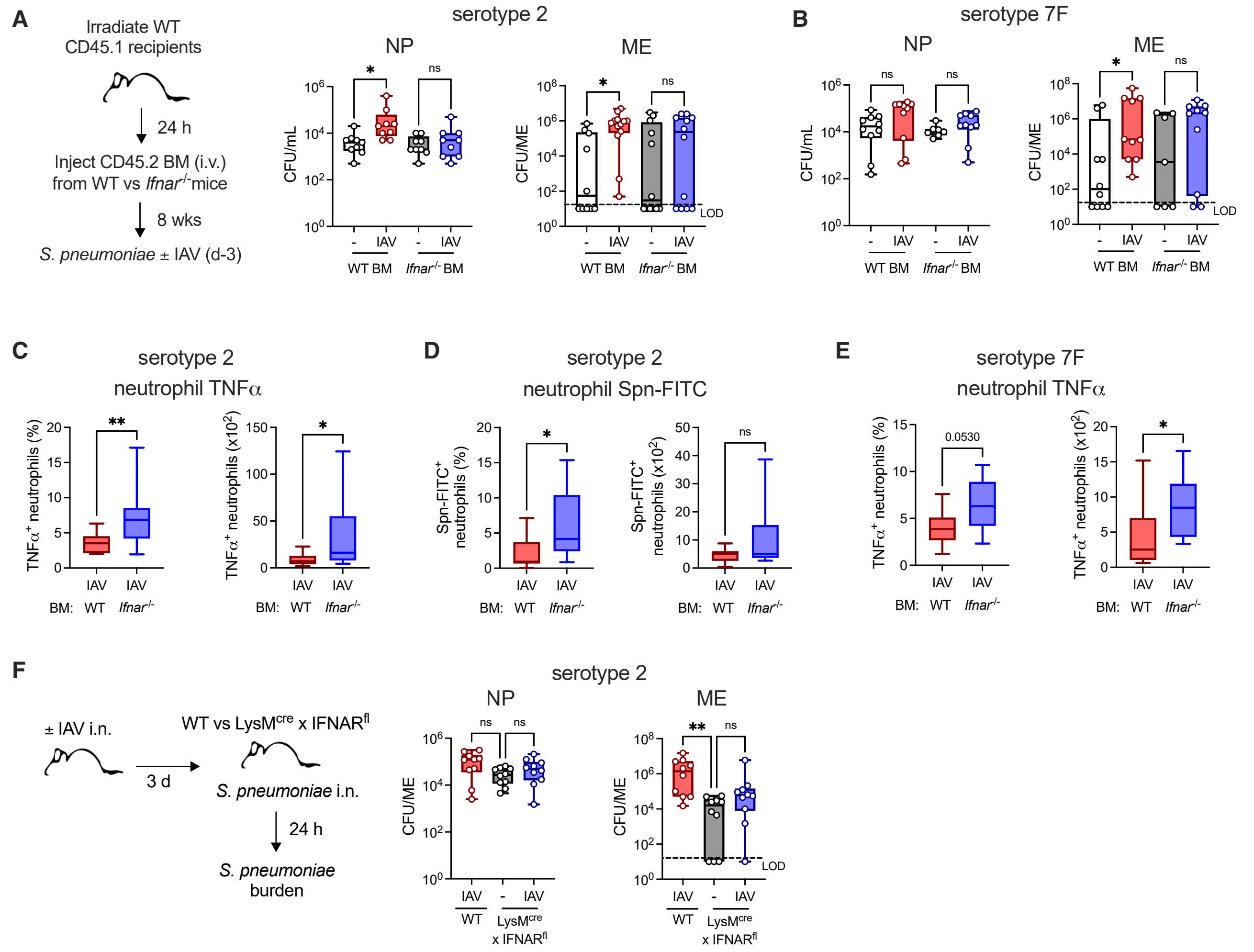

Figure 6. IFNAR signaling in irradiation-sensitive myeloid cells impairs neutrophil phagocytosis and pneumococcal clearance from the middle ear during viral co-infection

(A) Burdens of *S. pneumoniae* serotype 2 (10^7 CFU/mouse i.n.) at 24 hours post-infection detected in the nasopharynx (NP) and middle ear (ME) of irradiated CD45.1$^+$ WT recipients of CD45.2$^+$ WT or *Ifnar*$^{-/-}$ bone marrow with or without IAV co-infection (10^5 PFU/mouse i.n. 72 h prior to *S. pneumoniae* challenge), n = 10–13 mice/grp.

(B) Burdens of *S. pneumoniae* serotype 7F (10^5 CFU/mouse i.n.) at 24 h post-infection in irradiated recipients of WT or *Ifnar*$^{-/-}$ bone marrow with or without IAV co-infection, n = 7–10 mice/grp.

(C, D, and E) Percent and total number of TNFα$^+$ neutrophils and Spn-FITC$^+$ neutrophils, detected following 1 h incubation with FITC-labeled heat-killed *S. pneumoniae*, detected by flow cytometry in cells from the middle ear of IAV co-infected mice from (A). (E) Percent and total number of TNFα$^+$ neutrophils detected by flow cytometry in the middle ear of IAV co-infected mice from (B).

(F) Burden of *S. pneumoniae* serotype 7F in WT or LysM^{cre}xIFNAR^{flox} mice detected at 24 h post-infection with or without IAV co-infection, n = 10 mice/grp.

Data pooled from 3 independent experiments. Box boundaries indicate the 25th and 75th percentiles, with a horizontal line representing the median and whiskers indicating minimum and maximum values. LOD, limit of detection. *$p < 0.05$, **$p < 0.01$; Kruskal-Wallis with Dunn's post hoc test (A, B, and F) and Mann-Whitney U test (C, D, and E).

with *S. pneumoniae* serotype 7F, we noted a significant increase in middle ear levels of MPO, which is released during NETosis, compared to mice infected with *S. pneumoniae* alone (Figure 7A). To investigate the importance of NETs for poly(I:C)-enhanced *S. pneumoniae* middle ear infection, we compared infection in WT mice to mice deficient in peptidylarginine deiminase 4 (PAD4), an enzyme required for NET formation.[40] In the absence of poly(I:C) treatment, *S. pneumoniae* serotype 7F burdens in the nasopharynx and middle ear were similar between WT and PAD4-deficient mice (Figure S6A). However, in poly(I:C)-treated mice, *S. pneumoniae* burdens were significantly lower in PAD4-deficient mice compared to WT mice (Figure 7B). Nasopharyngeal burdens were similar between groups, as in mice not treated with poly(I:C) (Figure S6B). These data highlight NET generation as an important contributor to impaired *S. pneumoniae* clearance following type I IFN induction.

In PAD4-deficient mice, neutrophil survival in the middle ear during *S. pneumoniae* infection in poly(I:C)-treated mice was

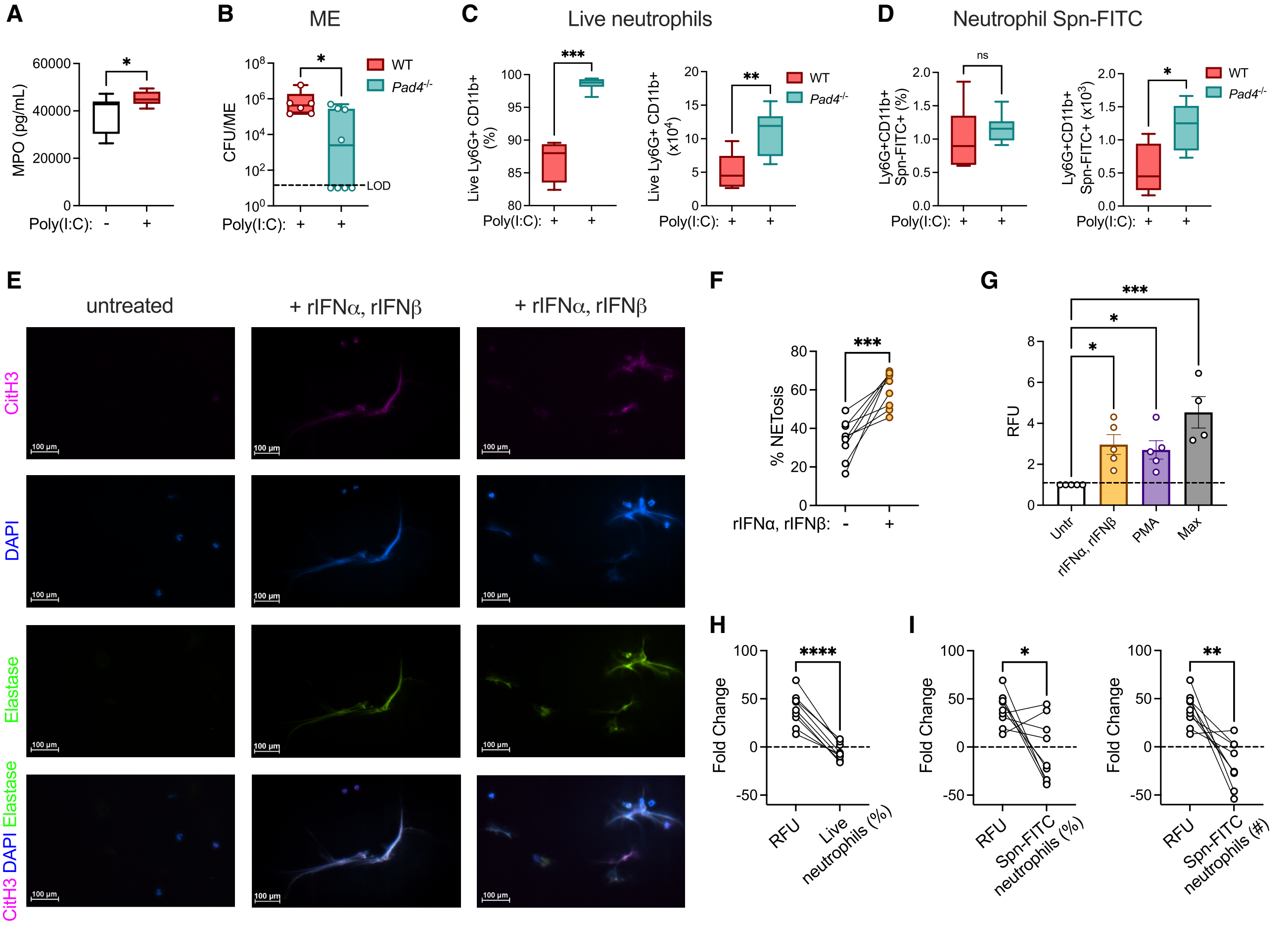

Figure 7. NETosis contributes to type I IFN impairment of *S. pneumoniae* clearance from the middle ear through the depletion of functional neutrophils

(A) Concentration of myeloperoxidase (MPO) detected in the middle ear of mice infected with *S. pneumoniae* serotype 7F (10^5 CFU/mouse i.n.) at 24 h post-infection with or without co-treatment with poly(I:C) (50 µg/mouse i.n.), n = 10 mice/grp.

(B) Burden of *S. pneumoniae* detected in WT or *Pad4*$^{-/-}$ mice at 24 h post-infection in mice co-treated with poly(I:C) (50 µg/mouse i.n.), n = 6–8 mice/grp.

(C and D) Percent and total number of live neutrophils detected by flow cytometry (C) and Spn-FITC^{+} neutrophils, detected following 1 h incubation with FITC-labeled heat-killed *S. pneumoniae* (D), in the middle ear of WT or *Pad4*$^{-/-}$ mice from (B).

(E) Representative immunohistochemistry images of NETs detected by citrullinated histone H3 (CitH3), DAPI, and elastase in neutrophils purified from the blood of healthy donors with or without exposure to recombinant IFNα2 and IFNβ (1 µg each/sample). Scale bars, 100 µm.

(F) Quantification of NETosis in samples from (E), n = 9 subjects.

(G) Relative fluorescent units (RFU) of SYTOX green detected in cultures of neutrophils purified from the blood of healthy donors with or without 3-h exposure to recombinant IFNα2 and IFNβ, PMA, or Triton X-100 detergent (Max), n = 5 subjects, plotted as mean ± SEM.

(H and I) Fold changes in RFU, percentage of live neutrophils, and percent and total number of Spn-FITC^{+} neutrophils detected following recombinant IFNα2 and IFNβ treatment relative to untreated controls in matched samples.

Data pooled from 3 (A–D), 4 (F), or 5 (H and I) independent experiments (F–I). Box boundaries indicate the 25th and 75th percentiles, with a horizontal line representing the median and whiskers indicating minimum and maximum values. LOD, limit of detection. *p <0 .05, **p <0 .01, ***p <0 .001, ****p <0 .0001; paired t test (F, H, and I), Mann-Whitney U test (A–D), and one-way ANOVA with Dunnett's post hoc test (G).

higher than in WT mice, as the percentage and total number of live neutrophils were significantly elevated (Figure 7C; Figure S6C). This finding suggests improved neutrophil survival during *S. pneumoniae* infection in *Pad4*$^{-/-}$ mice due to the deficiency in NET generation. To determine whether neutrophil phagocytosis was altered, uptake of FITC-labeled *S. pneumoniae* was compared in poly(I:C)-treated WT versus PAD4-deficient mice. In PAD4-deficient mice infected with *S. pneumoniae* and treated with poly(I:C), the total number of neutrophils that phagocytosed *S. pneumoniae* was significantly higher than in WT mice (Figure 7D). The number of TNFα+ neutrophils also trended higher in PAD4-deficient mice (Figure S6D). However, the percentage of phagocytic and TNFα+ neutrophils was similar regardless of genotype (Figure 7D; Figure S6D). These findings indicate improved neutrophil survival in the absence of NET formation following type I IFN induction, corresponding with greater numbers of active, phagocytic neutrophils.

To extend these findings to human neutrophils, NET formation was assessed in neutrophils isolated from the blood of healthy donors in the presence or absence of rIFNα2 and rIFNβ treatment. NETs were visualized by immunohistochemistry, with detection of long strands of extracellular DNA (DAPI) co-localized with citrullinated histone 3 (CitH3) and elastase in neutrophils treated with rIFNα2 and rIFNβ (Figure 7E). In contrast, NETs were not observed for untreated neutrophils. NETosis, quantified as the percentage of NETs over intact neutrophils, was significantly higher in matched samples treated with rIFNα2 and rIFNβ compared to untreated cells (Figure 7F). To validate these findings, NETosis was also quantified by SYTOX green, a cell-impermeable dye that detects extracellular DNA. NETosis quantified by SYTOX green staining was also significantly increased for human neutrophils treated with rIFNα2 and rIFNβ, compared to untreated neutrophils (Figure 7G). Type I IFN treatment induced a similar level of NETosis in neutrophils treated with phorbol 12-myristate 13-acetate (PMA) as a positive control for NET induction.

To evaluate the relationship between NETosis and neutrophil phagocytosis of *S. pneumoniae* following type I IFN stimulation, we assessed neutrophil viability and uptake of FITC-labeled *S. pneumoniae* in human neutrophils. Overall differences in neutrophil viability and *S. pneumoniae* uptake following treatment with rIFNα and rIFNβ were mixed, with trends toward decreased live neutrophils and *S. pneumoniae* uptake compared to untreated neutrophils (Figures S6E and S6F). However, consistent patterns emerged in matched samples analyzed for fold changes in NETosis compared to neutrophil viability and uptake of FITC-labeled *S. pneumoniae*. The type I IFN-induced increase in NETosis was paired with reductions in the percentage of live neutrophils detected by flow cytometry (Figure 7H), as anticipated following lytic NETosis. Neutrophils treated with rIFNα2 and rIFNβ also exhibited a reduced *S. pneumoniae* uptake response, indicated by a negative fold change in the number and percentage of FITC+ neutrophils across most samples, in contrast to the large increase in NETosis following type I IFN treatment (Figure 7I). Together, these data demonstrate the direct effects of type I IFNs on human neutrophil dysfunction, as type I IFN treatment strongly induced NETosis with corresponding defects in neutrophil viability and phagocytic capacity.

DISCUSSION

This study defines the immune signaling mechanisms underlying virus-induced hypersensitivity to bacterial replication in the middle ear, the most common infection experienced by children. Viral induction of type I IFN was sufficient to increase *S. pneumoniae* infection of the middle ear, aligning with a recent report indicating that poly(I:C) increased ear infection with NTHi and *M. catarrhalis*,[41] suggesting that this response enhances middle ear infection with all three major OM bacterial pathogens. Here, we define neutrophils as a critical target of type I IFN signaling, which directly impaired neutrophil activation and *S. pneumoniae* phagocytosis alongside loss of viable neutrophils through the induction of NETosis. These findings correspond with the strong clinical ties between viral infection and bacterial OM, where NETs are identified as the dominant host immune signature detected by proteomics in the middle ear of children with OM,[25] suggesting that type I IFN-mediated dysregulation of neutrophils is an important factor driving OM pathogenesis.

Our findings indicate that two hallmarks of IAV dysregulation of antibacterial defense in the lungs do not contribute to type I IFN enhancement of bacterial infection in the middle ear, namely, (1) the suppression of neutrophil recruitment and (2) the requirement for IFNγ. In the lungs, IAV co-infection is associated with changes in the function of alveolar macrophages (AMs), a resident macrophage population specific to lung alveoli.[42] Viral induction of T cell-derived IFNγ suppresses AM-mediated clearance of *S. pneumoniae*.[43,44] This pathway occurs upstream of the reported neutrophil recruitment defect, as AM depletion restored neutrophil recruitment to the lungs[45] and both neutrophil recruitment and *S. pneumoniae* clearance during lung IAV co-infection were improved in IFNγ receptor-deficient mice.[42] While the tissue-resident immune cell landscape of the middle ear is poorly defined, single-cell RNA sequencing in mice confirmed a population of Csf1r+ cells, which may represent tissue-resident macrophages.[46] The middle ear is embryogenically distinct from other tissues, arising from all three germ layers as well as the neural crest cells, resulting in a unique cellular architecture.[47] Epithelial cells contribute to immune programming in tissue-resident macrophage populations including AMs.[48,49] Within the ear, immune activation must be balanced with protection of the vestibulocochlear system, where damage causes both sensorineural hearing loss and degradation of the balance and spatial orientation system.[47] The differential effects of type I IFN signaling in the middle ear versus lungs may, therefore, be a consequence of distinct tissue-resident macrophage composition or the impact of alternative macrophage programming by the middle ear epithelium.

While viral impairment of *S. pneumoniae* clearance is multifactorial,[50] the cell-specific targets responsible for impaired bacterial clearance following viral immune dysregulation are poorly defined. In separating the importance of epithelial IFNAR for viral control from the other effects of IFNAR signaling, we found that IFNAR signaling on hematopoietic cells impaired bacterial clearance and neutrophil antibacterial defense. These findings offer a striking contrast to a recent report using a near-identical experimental system that found that irradiation-resistant cells, likely AMs, were responsible for IFNAR-mediated impairment of bacterial clearance during IAV co-infection with *S. pneumoniae* in the lungs, with a minimal role for neutrophils in this setting.[51] Together, these findings delineate an important contribution for IFNAR signaling on hematopoietic cells to IAV-mediated dysregulation of antibacterial defense in the middle ear.

Type I IFN increased neutrophil NETosis, consistent with other reports,[52–55] which was associated with reduced middle ear neutrophil survival. In people with chronically elevated IFN, neutrophils undergo spontaneous NETosis.[56] Clinically, NETs are abundant in the middle ear.[25] While intact neutrophils were detected in middle ear fluid samples from children with OM, their phagocytic capacity was significantly lower than that of circulating neutrophils. In mice, blocking NET induction rescued effective bacterial clearance in the presence of type I IFN, primarily by improving neutrophil survival, resulting in higher numbers of active, phagocytic neutrophils. Neutrophil TNFα

signaling, including cell-intrinsic signaling through membrane-bound TNFα binding TNFR2, contributes to the regulation of NETosis, with the timing of TNFα exposure dictating either reduced or increased NETosis.[57] *S. pneumoniae* encodes endonucleases that cleave NETs, reducing their effectiveness.[39] In addition to *S. pneumoniae*, the other predominant OM bacterial pathogens encode proteins enabling escape of NET-mediated killing, including the DNAIIB protein HU produced by NTHi and the nuclease NucM produced by *M. catarrhalis*.[58,59] Each of these bacteria were reported in middle ear samples from children with recurrent OM, with live bacteria noted in most samples tested, all of which had extensive NETs.[60] Overall, these findings suggest that strategies to block NET formation may improve middle ear clearance of *S. pneumoniae* and potentially other OM bacterial pathogens, by neutrophils.

We focused on the consequences of virus-induced type I IFN on cellular responses within the middle ear, as type I IFN induction was sufficient to increase middle ear bacterial burdens in mice without significant effects in the nasopharynx. The lack of difference in nasopharyngeal burdens suggests that the mechanism of poly(I:C) enhancement is tied to an improved capacity for *S. pneumoniae* invasion, adherence, or survival in the middle ear, rather than a higher burden in the nasopharynx simply seeding more bacteria to the middle ear cavity. The type I IFN-driven increase in middle ear NETs may contribute to this differential infection success, as NETs are not a reported component of the transient neutrophil influx observed during *S. pneumoniae* colonization in humans or mice.[61,62] IAV-induced type I IFN was previously reported to suppress macrophage recruitment to the nasopharynx without affecting neutrophils during *S. pneumoniae* colonization.[63] In the middle ear, we observed similar macrophage recruitment during *S. pneumoniae* infection regardless of IAV co-infection. While neutrophil phagocytosis was not directly assessed in the nasopharynx, these findings imply distinct consequences of virus-induced type I IFN on *S. pneumoniae* colonization versus middle ear infection.

In the clinical cohort, dominance of the top three major bacterial OM pathogens in the upper airway was paired with unexpectedly high carriage of respiratory viral pathogens, which was greater than that reported for children with symptomatic upper respiratory infections.[27] Consistent with our detection of elevated inflammatory cytokines during active infection, other reports indicate higher pro-inflammatory cytokine levels in middle ear fluid samples with bacterial and/or viral pathogens present.[64,65] While we did not assess nasopharyngeal inflammation, Watkins et al. reported that colonization with bacterial pathogens correlated with increased nasopharyngeal levels of TNFα, IL-1β, and IL-6.[27] Viral load in the nasopharynx was also associated with an elevated tissue IFN response, with significantly higher IP-10 in children carrying multiple viral pathogens.[27] Another report identified a subset of ‘‘IFN-stimulated’’ neutrophils recruited to the nasal mucosa following IAV infection in mice.[66] In the mouse model, poly(I:C) induction of type I IFN significantly elevated levels of IP-10 (CXCL10), TNFα, and IL-6 in the middle ear, correlating with the increased IP-10 (CXCL10) and IL-6 detected in the middle ear of children with OM during active infection and with higher viral load, when TNFα was also significantly higher. Collectively, these studies point to a potential protective effect for viral targeting through vaccines or anti-viral therapy to reduce OM incidence. While not the current standard of care, treatment with oseltamivir in children with laboratory-confirmed influenza virus infection significantly reduced OM incidence,[67,68] the mechanism of which we propose relates to mitigation of virus-mediated impairment of defense against bacterial middle ear infections.

Together, these findings delineate the immune modulatory effects of viral co-infection on antibacterial defense in the middle ear. In contrast to the lungs, the deleterious effects of virus-induced type I IFN signaling were restrained to hematopoietic cells, with a significant impairment of neutrophil activation and phagocytic function alongside loss of viable neutrophils through induction of NETosis. Overall, these data highlight neutrophil activation as an important therapeutic target to interfere with virus-mediated impairment of antibacterial defense in children with OM.

Limitations of the study

Here we modeled acute OM, whereas a significant portion of the clinical burden occurs in children with recurrent or chronic OM. However, signaling pathways initiated during OM onset may direct future outcomes after resolution of acute infection. The sustained presence of neutrophils and NETs in clinical OM samples highlights the importance of understanding the long-term impact of viral co-infection on this cell type. Another caveat of our model is the use of adult mice, while children under the age of 5 encompass most of the OM burden. Immunologically, specific pathogen-free mice have an underdeveloped immune system compared to wild-caught mice, which have more similar tissue-resident immune cell compositions as adult humans, alongside improved infection defense.[69,70] In children, several immune responses including TLR signaling and myeloid cell activation are underdeveloped.[71,72] While direct comparisons are limited, these studies point to immunologic analogs in adult specific-pathogen-free mice with the immature immune system in children. However, there is also evidence for extended immune maturation delay in OM-prone children, including lower myeloid cell responsiveness to *S. pneumoniae*.[73–75] These observations highlight the need to address the underlying microbial and immune factors predisposing to chronic and recurrent OM in children.

RESOURCE AVAILABILITY

Lead contact

Further information and requests for resources and reagents should be directed to and will be fulfilled by the lead contact, Sarah E. Clark (sarah.e.clark@cuanschutz.edu).

Materials availability

This study did not generate unique reagents.

Data and code availability

- Sequencing data generated in this study are available in the NCBI Short Read Archive under BioProject: PRJNA1404333. All other data are included in the manuscript figures and supplemental information. Raw data used to generate all figures are available from the lead contact upon request.
- This paper does not report original code.

- Any additional information required to reanalyze the data reported in this paper is available from the lead contact upon request.

ACKNOWLEDGMENTS

We acknowledge valuable input on this project from our colleagues in the Department of Otolaryngology – Head & Neck Surgery. We thank Dr. Jenna Guthmiller for providing IAV x31 for these studies. We also thank Dr. Jason Rosch for providing *S. pneumoniae* serotype 7F and 19F strains for this study. This study was supported by the National Institute of Deafness and Communications Disorders (NIDCD) of the National Institutes of Health (NIH) award number R21DC019169 (S.E.C.) with additional support from the NIDCD, NIH Institutional Training in Otolaryngology Research award number T32DC012280 (S.C.S.).

AUTHOR CONTRIBUTIONS

Conceptualization, S.E.C., S.A.G., and S.C.S.; methodology, S.C.S., T.L.J., and G.H.; data curation, S.C.S., T.L.J., G.H., J.T.F., and W.J.; formal analysis, S.C.S., B.P.L., Z.D., J.K.H., and S.A.G.; writing – original draft, S.E.C. and S.C.S.; writing – review and editing, all authors.

DECLARATION OF INTERESTS

The authors declare no competing interests.

STAR★METHODS

Detailed methods are provided in the online version of this paper and include the following:

- KEY RESOURCES TABLE
- EXPERIMENTAL MODEL AND STUDY PARTICIPANT DETAILS
  - Human subjects
  - Animals
  - Bacteria
  - Viruses
- METHOD DETAILS
  - High-throughput DNA sequencing for microbiome analysis
  - Human neutrophil isolation
  - Mouse infection
  - Bone marrow chimeras
  - Flow cytometry
  - Neutrophil functional assays
  - Cytokine and chemokine analysis
  - Histology
- QUANTIFICATION AND STATISTICAL ANALYSIS

SUPPLEMENTAL INFORMATION

Supplemental information can be found online at https://doi.org/10.1016/j.xcrm.2026.102846.

Received: February 10, 2026
Revised: April 2, 2026
Accepted: May 11, 2026
Published: June 3, 2026

REFERENCES

1. Meherali, S., Campbell, A., Hartling, L., and Scott, S. (2019). Understanding Parents' Experiences and Information Needs on Pediatric Acute Otitis Media: A Qualitative Study. J. Patient Exp. *6*, 53–61.
2. Monasta, L., Ronfani, L., Marchetti, F., Montico, M., Vecchi Brumatti, L., Bavcar, A., Grasso, D., Barbiero, C., and Tamburlini, G. (2012). Burden of Disease Caused by Otitis Media: Systematic Review and Global Estimates. PLoS One *7*, e36226.
3. Qureishi, A., Lee, Y., Belfield, K., Birchall, J., and Daniel, M. (2014). Update on otitis media – prevention and treatment. Infect. Drug Resist. *15–10*.
4. Yildirim-Baylan, M., Schachern, P., Tsuprun, V., Shiabata, D., Paparella, M.M., and Cureoglu, S. (2014). The pathology of silent otitis media: A predecessor to tympanogenic meningitis in infants. Int. J. Pediatr. Otorhinolaryngol. *78*, 451–454.
5. Venekamp, R.P., Sanders, S.L., Glasziou, P.P., Del Mar, C.B., and Rovers, M.M. (2015). Antibiotics for acute otitis media in children. Cochrane Database Syst. Rev. *2015*, CD000219.
6. Rosenfeld, R.M., Tunkel, D.E., Schwartz, S.R., Anne, S., Bishop, C.E., Chelius, D.C., Hackell, J., Hunter, L.L., Keppel, K.L., Kim, A.H., et al. (2022). Clinical Practice Guideline: Tympanostomy Tubes in Children (Update). Otolaryngol. Head Neck Surg. *166*, S1–S55.
7. Sillanpää, S., et al. (2017). Next-Generation Sequencing Combined with Specific PCR Assays To Determine the Bacterial 16S rRNA Gene Profiles of Middle Ear Fluid Collected from Children with Acute Otitis Media. mSphere *2*. https://doi.org/10.1128/msphere.00006-17.
8. Bergenfelz, C., and Hakansson, A.P. (2017). Streptococcus pneumoniae Otitis Media Pathogenesis and How It Informs Our Understanding of Vaccine Strategies. Curr. Otorhinolaryngol. Rep. *5*, 115–124.
9. Nokso-Koivisto, J., Marom, T., and Chonmaitree, T. (2015). Importance of viruses in acute otitis media. Curr. Opin. Pediatr. *27*, 110–115.
10. Chonmaitree, T., Revai, K., Grady, J.J., Clos, A., Patel, J.A., Nair, S., Fan, J., and Henrickson, K.J. (2008). Viral Upper Respiratory Tract Infection and Otitis Media Complication in Young Children. Clin. Infect. Dis. *46*, 815–823.
11. Winther, B., Doyle, W.J., and Alper, C.M. (2006). A high prevalence of new onset otitis media during parent diagnosed common colds. Int. J. Pediatr. Otorhinolaryngol. *70*, 1725–1730.
12. Chonmaitree, T., Trujillo, R., Jennings, K., Alvarez-Fernandez, P., Patel, J.A., Loeffelholz, M.J., Nokso-Koivisto, J., Matalon, R., Pyles, R.B., Miller, A.L., and McCormick, D.P. (2016). Acute Otitis Media and Other Complications of Viral Respiratory Infection. Pediatrics *137*, e20153555.
13. Chonmaitree, T., Ruohola, A., and Hendley, J.O. (2012). Presence of Viral Nucleic Acids in the Middle Ear: Acute Otitis Media Pathogen or Bystander? Pediatr. Infect. Dis. J. *31*, 325–330.
14. Sawada, S., Okutani, F., and Kobayashi, T. (2019). Comprehensive Detection of Respiratory Bacterial and Viral Pathogens in the Middle Ear Fluid and Nasopharynx of Pediatric Patients With Acute Otitis Media. Pediatr. Infect. Dis. J. *38*, 1199–1203.
15. Tamir, S.O., Bialasiewicz, S., Brennan-Jones, C.G., Der, C., Kariv, L., Macharia, I., Marsh, R.L., Seguya, A., and Thornton, R. (2023). ISOM 2023 research Panel 4 - Diagnostics and microbiology of otitis media. Int. J. Pediatr. Otorhinolaryngol. *174*, 111741.
16. Manning, J., Dunne, E.M., Wang, N., Pedersen, J.S., Ogier, J.M., Burt, R.A., Mulholland, E.K., Robins-Browne, R.M., Malley, R., Wijburg, O.L., and Satzke, C. (2019). Effect of a pneumococcal whole cell vaccine on influenza A-induced pneumococcal otitis media in infant mice. Vaccine *37*, 3495–3504.
17. Short, K.R., Diavatopoulos, D.A., Thornton, R., Pedersen, J., Strugnell, R.A., Wise, A.K., Reading, P.C., and Wijburg, O.L. (2011). Influenza Virus Induces Bacterial and Nonbacterial Otitis Media. J. Infect. Dis. *204*, 1857–1865.
18. Chiavolini, D., Pozzi, G., and Ricci, S. (2008). Animal Models of Streptococcus pneumoniae Disease. Clin. Microbiol. Rev. *21*, 666–685.
19. Wren, J.T., Blevins, L.K., Pang, B., King, L.B., Perez, A.C., Murrah, K.A., Reimche, J.L., Alexander-Miller, M.A., and Swords, W.E. (2014). Influenza A Virus Alters Pneumococcal Nasal Colonization and Middle Ear Infection Independently of Phase Variation. Infect. Immun. *82*, 4802–4812.
20. Cao, J., Wang, D., Xu, F., Gong, Y., Wang, H., Song, Z., Li, D., Zhang, H., Li, D., Zhang, L., et al. (2014). Activation of IL-27 signalling promotes

development of postinfluenza pneumococcal pneumonia. EMBO Mol. Med. *6*, 120–140.

21. Shahangian, A., Chow, E.K., Tian, X., Kang, J.R., Ghaffari, A., Liu, S.Y., Belperio, J.A., Cheng, G., and Deng, J.C. (2009). Type I IFNs mediate development of postinfluenza bacterial pneumonia in mice. J. Clin. Investig. *119*, 1910–1920.
22. Li, W., Moltedo, B., and Moran, T.M. (2012). Type I Interferon Induction during Influenza Virus Infection Increases Susceptibility to Secondary Streptococcus pneumoniae Infection by Negative Regulation of γδ T Cells. J. Virol. *86*, 12304–12312.
23. Narayana Moorthy, A., Narasaraju, T., Rai, P., Perumalsamy, R., Tan, K.B., Wang, S., Engelward, B., and Chow, V.T.K. (2013). In vivo and in vitro studies on the roles of neutrophil extracellular traps during secondary pneumococcal pneumonia after primary pulmonary influenza infection. Front. Immunol. *4*, 56.
24. Narasaraju, T., Yang, E., Samy, R.P., Ng, H.H., Poh, W.P., Liew, A.A., Phoon, M.C., van Rooijen, N., and Chow, V.T. (2011). Excessive Neutrophils and Neutrophil Extracellular Traps Contribute to Acute Lung Injury of Influenza Pneumonitis. Am. J. Pathol. *179*, 199–210.
25. Val, S., Poley, M., Brown, K., Choi, R., Jeong, S., Colberg-Poley, A., Rose, M.C., Panchapakesan, K.C., Devaney, J.C., Perez-Losada, M., and Preciado, D. (2016). Proteomic Characterization of Middle Ear Fluid Confirms Neutrophil Extracellular Traps as a Predominant Innate Immune Response in Chronic Otitis Media. PLoS One *11*, e0152865.
26. Reid, S.D., Hong, W., Dew, K.E., Winn, D.R., Pang, B., Watt, J., Glover, D.T., Hollingshead, S.K., and Swords, W.E. (2009). Streptococcus pneumoniae Forms Surface-Attached Communities in the Middle Ear of Experimentally Infected Chinchillas. J. Infect. Dis. *199*, 786–794.
27. Watkins, T.A., Green, A.B., Amat, J.A.R., Cheemarla, N.R., Hänsel, K., Lozano, R., Dudgeon, S.N., Germain, G., Landry, M.L., Schulz, W.L., and Foxman, E.F. (2024). High burden of viruses and bacterial pathobionts drives heightened nasal innate immunity in children. J. Exp. Med. *221*, e20230911.
28. Galanti, M., Birger, R., Ud-Dean, M., Filip, I., Morita, H., Comito, D., Anthony, S., Freyer, G.A., Ibrahim, S., Lane, B., et al. (2019). Rates of asymptomatic respiratory virus infection across age groups. Epidemiol. Infect. *147*, e176.
29. Buttmann, M., Berberich-Siebelt, F., Serfling, E., and Rieckmann, P. (2007). Interferon-beta is a potent inducer of interferon regulatory factor-1/2-dependent IP-10/CXCL10 expression in primary human endothelial cells. J. Vasc. Res. *44*, 51–60.
30. Jochems, S.P., Marcon, F., Carniel, B.F., Holloway, M., Mitsi, E., Smith, E., Gritzfeld, J.F., Solórzano, C., Reiné, J., Pojar, S., et al. (2018). Inflammation induced by influenza virus impairs human innate immune control of pneumococcus. Nat. Immunol. *19*, 1299–1308.
31. Mommert-Tripon, M., Parraud, D., Grosbois, C., Gaymard, A., Cheynet, V., Lina, B., Oriol, G., Laurent, F., Dupré, C., Semanas, Q., et al. (2024). Advancing respiratory virus diagnostics: integrating the nasal IFN-I score for improved viral detection. EBioMedicine *110*, 105450.
32. Cheemarla, N.R., Hanron, A., Fauver, J.R., Bishai, J., Watkins, T.A., Brito, A.F., Zhao, D., Alpert, T., Vogels, C.B.F., Ko, A.I., et al. (2023). Nasal host response-based screening for undiagnosed respiratory viruses: a pathogen surveillance and detection study. Lancet Microbe *4*, e38–e46.
33. Casey, J.R., Adlowitz, D.G., and Pichichero, M.E. (2010). New Patterns in the Otopathogens Causing Acute Otitis Media Six to Eight Years After Introduction of Pneumococcal Conjugate Vaccine. Pediatr. Infect. Dis. J. *29*, 304–309.
34. Ekinci, E., Desmet, S., Van Heirstraeten, L., Mertens, C., Wouters, I., Beutels, P., Verhaegen, J., Malhotra-Kumar, S., and Theeten, H.; NPcarriage Group (2021). Streptococcus pneumoniae Serotypes Carried by Young Children and Their Association With Acute Otitis Media During the Period 2016–2019. Front. Pediatr. *9*, 664083.
35. Lanie, J.A., Ng, W.L., Kazmierczak, K.M., Andrzejewski, T.M., Davidsen, T.M., Wayne, K.J., Tettelin, H., Glass, J.I., and Winkler, M.E. (2007). Genome Sequence of Avery's Virulent Serotype 2 Strain D39 of Streptococcus pneumoniae and Comparison with That of Unencapsulated Laboratory Strain R6. J. Bacteriol. *189*, 38–51.
36. Janoff, E.N., Fasching, C., Orenstein, J.M., Rubins, J.B., Opstad, N.L., and Dalmasso, A.P. (1999). Killing of Streptococcus pneumoniae by capsular polysaccharide–specific polymeric IgA, complement, and phagocytes. J. Clin. Investig. *104*, 1139–1147.
37. Pinto, A.K., Brien, J.D., Lam, C.Y.K., Johnson, S., Chiang, C., Hiscott, J., Sarathy, V.V., Barrett, A.D., Shresta, S., and Diamond, M.S. (2015). Defining New Therapeutics Using a More Immunocompetent Mouse Model of Antibody-Enhanced Dengue Virus Infection. mBio *6*, e01316. https://doi.org/10.1128/mbio.01316-15.
38. Diamond, M.S., Kinder, M., Matsushita, H., Mashayekhi, M., Dunn, G.P., Archambault, J.M., Lee, H., Arthur, C.D., White, J.M., Kalinke, U., et al. (2011). Type I interferon is selectively required by dendritic cells for immune rejection of tumors. J. Exp. Med. *208*, 1989–2003.
39. Beiter, K., Wartha, F., Albiger, B., Normark, S., Zychlinsky, A., and Henriques-Normark, B. (2006). An Endonuclease Allows Streptococcus pneumoniae to Escape from Neutrophil Extracellular Traps. Curr. Biol. *16*, 401–407.
40. Sørensen, O.E., and Borregaard, N. (2016). Neutrophil extracellular traps — the dark side of neutrophils. J. Clin. Investig. *126*, 1612–1620.
41. Echlin, H., Iverson, A., McKnight, A., and Rosch, J.W. (2024). A Trivalent Live Vaccine Elicits Cross-Species Protection Against Acute Otitis Media in a Murine Model. Vaccines *12*, 1432.
42. Palani, S., Uddin, M.B., McKelvey, M., Shao, S., and Sun, K. (2023). Immune predisposition drives susceptibility to pneumococcal pneumonia after mild influenza A virus infection in mice. Front. Immunol. *14*, 1272920.
43. Sun, K., and Metzger, D.W. (2008). Inhibition of pulmonary antibacterial defense by interferon-γ during recovery from influenza infection. Nat. Med. *14*, 558–564.
44. Roberts, S., Salmon, S.L., Steiner, D.J., Williams, C.M., Metzger, D.W., and Furuya, Y. (2019). Allergic Airway Disease Prevents Lethal Synergy of Influenza A Virus-Streptococcus pneumoniae Coinfection. mBio *10*, e01335-19. https://doi.org/10.1128/mbio.01335-19.
45. Didierlaurent, A., Goulding, J., Patel, S., Snelgrove, R., Low, L., Bebien, M., Lawrence, T., van Rijt, L.S., Lambrecht, B.N., Sirard, J.C., and Hussell, T. (2008). Sustained desensitization to bacterial Toll-like receptor ligands after resolution of respiratory influenza infection. J. Exp. Med. *205*, 323–329.
46. Ryan, A.F., Nasamran, C.A., Pak, K., Draf, C., Fisch, K.M., Webster, N., and Kurabi, A. (2020). Single-Cell Transcriptomes Reveal a Complex Cellular Landscape in the Middle Ear and Differential Capacities for Acute Response to Infection. Front. Genet. *11*, 358.
47. Anthwal, N., and Thompson, H. (2016). The development of the mammalian outer and middle ear. J. Anat. *228*, 217–232.
48. Schneider, C., Nobs, S.P., Kurrer, M., Rehrauer, H., Thiele, C., and Kopf, M. (2014). Induction of the nuclear receptor PPAR-γ by the cytokine GM-CSF is critical for the differentiation of fetal monocytes into alveolar macrophages. Nat. Immunol. *15*, 1026–1037.
49. Bonnardel, J., T'Jonck, W., Gaublomme, D., Browaeys, R., Scott, C.L., Martens, L., Vanneste, B., De Prijck, S., Nedospasov, S.A., Kremer, A., et al. (2019). Stellate Cells, Hepatocytes, and Endothelial Cells Imprint the Kupffer Cell Identity on Monocytes Colonizing the Liver Macrophage Niche. Immunity *51*, 638–654.e9.
50. Oliva, J., and Terrier, O. (2021). Viral and Bacterial Co-Infections in the Lungs: Dangerous Liaisons. Viruses *13*, 1725.
51. Palani, S., Uddin, M.B., McKelvey, M., Shao, S., Wu, W., Bao, X., Sun, J., and Sun, K. (2025). Type I Interferon Targets Alveolar Macrophages to Promote Bacterial Pneumonia after Viral Infection. Am. J. Respir. Cell Mol. Biol. *73*, 264–274.

CellPress
OPEN ACCESS

52. Garcia-Romo, G.S., Caielli, S., Vega, B., Connolly, J., Allantaz, F., Xu, Z., Punaro, M., Baisch, J., Guiducci, C., Coffman, R.L., et al. (2011). Netting Neutrophils Are Major Inducers of Type I IFN Production in Pediatric Systemic Lupus Erythematosus. Sci. Transl. Med. *3*, 73ra20.

53. Ma, Y., Wang, M., Jia, J., Meng, J., Teng, J., Zhu, D., Shi, H., Sun, Y., Su, Y., Liu, H., et al. (2022). Enhanced type I interferon signature induces neutrophil extracellular traps enriched in mitochondrial DNA in adult-onset Still's disease. J. Autoimmun. *127*, 102793.

54. Moreira-Teixeira, L., Stimpson, P.J., Stavropoulos, E., Hadebe, S., Chakravarty, P., Ioannou, M., Aramburu, I.V., Herbert, E., Priestnall, S.L., Suarez-Bonnet, A., et al. (2020). Type I IFN exacerbates disease in tuberculosis-susceptible mice by inducing neutrophil-mediated lung inflammation and NETosis. Nat. Commun. *11*, 5566.

55. Grunwell, J.R., Stephenson, S.T., Mohammad, A.F., Jones, K., Mason, C., Opolka, C., and Fitzpatrick, A.M. (2020). Differential type I interferon response and primary airway neutrophil extracellular trap release in children with acute respiratory distress syndrome. Sci. Rep. *10*, 19049.

56. Gul, E., Sayar, E.H., Gungor, B., Eroglu, F.K., Surucu, N., Keles, S., Guner, S.N., Findik, S., Alpdundar, E., Ayanoglu, I.C., et al. (2018). Type I IFN–related NETosis in ataxia telangiectasia and Artemis deficiency. J. Allergy Clin. Immunol. *142*, 246–257.

57. Neuenfeldt, F., Schumacher, J.C., Grieshaber-Bouyer, R., Habicht, J., Schröder-Braunstein, J., Gauss, A., Merle, U., Niesler, B., Heineken, N., Dalpke, A., et al. (2022). Inflammation induces pro-NETotic neutrophils via TNFR2 signaling. Cell Rep. *39*, 110710.

58. Buzzo, J.R., Devaraj, A., Gloag, E.S., Jurcisek, J.A., Robledo-Avila, F., Kesler, T., Wilbanks, K., Mashburn-Warren, L., Balu, S., Wickham, J., et al. (2021). Z-form extracellular DNA is a structural component of the bacterial biofilm matrix. Cell *184*, 5740–5758.e17.

59. Tan, A., Li, W.S., Verderosa, A.D., Blakeway, L.V., D Mubaiwa, T., Totsika, M., and Seib, K.L. (2019). Moraxella catarrhalis NucM is an entry nuclease involved in extracellular DNA and RNA degradation, cell competence and biofilm scaffolding. Sci. Rep. *9*, 2579.

60. Thornton, R.B., Wiertsema, S.P., Kirkham, L.A.S., Rigby, P.J., Vijayasekaran, S., Coates, H.L., and Richmond, P.C. (2013). Neutrophil Extracellular Traps and Bacterial Biofilms in Middle Ear Effusion of Children with Recurrent Acute Otitis Media – A Potential Treatment Target. PLoS One *8*, e53837.

61. Matthias, K.A., Roche, A.M., Standish, A.J., Shchepetov, M., and Weiser, J.N. (2008). Neutrophil-Toxin Interactions Promote Antigen Delivery and Mucosal Clearance of Streptococcus pneumoniae. J. Immunol. *180*, 6246–6254.

62. Nikolaou, E., Jochems, S.P., Mitsi, E., Pojar, S., Blizard, A., Reiné, J., Solórzano, C., Negera, E., Carniel, B., Soares-Schanoski, A., et al. (2021). Experimental Human Challenge Defines Distinct Pneumococcal Kinetic Profiles and Mucosal Responses between Colonized and Non-Colonized Adults. mBio *12*, e02020-20. https://doi.org/10.1128/mbio.02020-20.

63. Nakamura, S., Davis, K.M., and Weiser, J.N. (2011). Synergistic stimulation of type I interferons during influenza virus coinfection promotes Streptococcus pneumoniae colonization in mice. J. Clin. Investig. *121*, 3657–3665.

64. Skovbjerg, S., Roos, K., Nowrouzian, F., Lindh, M., Holm, S.E., Adlerberth, I., Olofsson, S., and Wold, A.E. (2010). High cytokine levels in perforated acute otitis media exudates containing live bacteria. Clin. Microbiol. Infect. *16*, 1382–1388.

65. Skovbjerg, S., Roos, K., Andersson, M., Rabe, H., Nilsson, S., Lindh, M., and Wold, A.E. (2020). Inflammatory Mediator Profiles in Secretory Otitis Media in Relationship to Viable Bacterial Pathogens and Bacterial and Viral Nucleic Acids. J. Interf. Cytokine Res. *40*, 555–569.

66. Kazer, S.W., Match, C.M., Langan, E.M., Messou, M.A., LaSalle, T.J., O'Leary, E., Marbourg, J., Naughton, K., von Andrian, U.H., and Ordovas-Montanes, J. (2024). Primary nasal influenza infection rewires tissue-scale memory response dynamics. Immunity *57*, 1955–1974.e8.

67. WHITLEY, R.J., Hayden, F.G., Reisinger, K.S., Young, N., Dutkowski, R., Ipe, D., Mills, R.G., and Ward, P. (2001). Oral oseltamivir treatment of influenza in children. Pediatr. Infect. Dis. J. *20*, 127–133.

68. Winther, B., Block, S.L., Reisinger, K., and Dutkowski, R. (2010). Impact of oseltamivir treatment on the incidence and course of acute otitis media in children with influenza. Int. J. Pediatr. Otorhinolaryngol. *74*, 684–688.

69. Beura, L.K., Hamilton, S.E., Bi, K., Schenkel, J.M., Odumade, O.A., Casey, K.A., Thompson, E.A., Fraser, K.A., Rosato, P.C., Filali-Mouhim, A., et al. (2016). Normalizing the environment recapitulates adult human immune traits in laboratory mice. Nature *532*, 512–516.

70. Rosshart, S.P., Vassallo, B.G., Angeletti, D., Hutchinson, D.S., Morgan, A.P., Takeda, K., Hickman, H.D., McCulloch, J.A., Badger, J.H., Ajami, N.J., et al. (2017). Wild Mouse Gut Microbiota Promotes Host Fitness and Improves Disease Resistance. Cell *171*, 1015–1028.e13.

71. Khan, M.N., and Pichichero, M.E. (2014). The host immune dynamics of pneumococcal colonization: Implications for novel vaccine development. Hum. Vaccin. Immunother. *10*, 3688–3699.

72. Kollmann, T.R., Levy, O., Montgomery, R.R., and Goriely, S. (2012). Innate Immune Function by Toll-like Receptors: Distinct Responses in Newborns and the Elderly. Immunity *37*, 771–783.

73. Basha, S., Kaur, R., Mosmann, T.R., and Pichichero, M.E. (2017). Reduced T-Helper 17 Responses to Streptococcus pneumoniae in Infection-Prone Children Can Be Rescued by Addition of Innate Cytokines. J. Infect. Dis. *215*, 1321–1330.

74. Pichichero, M.E., Casey, J.R., and Almudevar, A. (2013). Nonprotective Responses to Pediatric Vaccines Occur in Children Who Are Otitis Prone. Pediatr. Infect. Dis. J. *32*, 1163–1168.

75. Martino, D., Schultz, N., Kaur, R., van Haren, S.D., Kresoje, N., Hoch, A., Diray-Arce, J., Su, J.L., Levy, O., and Pichichero, M.; in association with the IDEAL Consortium (2024). Respiratory infection- and asthma-prone, low vaccine responder children demonstrate distinct mononuclear cell DNA methylation pathways. Clin. Epigenetics *16*, 85.

76. Edgar, R.C., Haas, B.J., Clemente, J.C., Quince, C., and Knight, R. (2011). UCHIME improves sensitivity and speed of chimera detection. Bioinformatics *27*, 2194–2200.

77. Ewing, B., and Green, P. (1998). Base-Calling of Automated Sequencer Traces Using Phred. II. Error Probabilities. Genome Res. *8*, 186–194.

78. Ewing, B., Hillier, L., Wendl, M.C., and Green, P. (1998). Base-Calling of Automated Sequencer Traces UsingPhred. I. Accuracy Assessment. Genome Res. *8*, 175–185.

79. Zafar, M.A., Kono, M., Wang, Y., Zangari, T., and Weiser, J.N. (2016). Infant Mouse Model for the Study of Shedding and Transmission during Streptococcus pneumoniae Monoinfection. Infect. Immun. *84*, 2714–2722.

80. McCullers, J.A., McAuley, J.L., Browall, S., Iverson, A.R., Boyd, K.L., and Henriques Normark, B. (2010). Influenza Enhances Susceptibility to Natural Acquisition of and Disease due to Streptococcus pneumoniae in Ferrets. J. Infect. Dis. *202*, 1287–1295.

81. Rutigliano, J.A., Sharma, S., Morris, M.Y., Oguin, T.H., 3rd, McClaren, J.L., Doherty, P.C., and Thomas, P.G. (2014). Highly Pathological Influenza A Virus Infection Is Associated with Augmented Expression of PD-1 by Functionally Compromised Virus-Specific CD8+ T Cells. J. Virol. *88*, 1636–1651.

82. Hara, N., Alkanani, A.K., Ir, D., Robertson, C.E., Wagner, B.D., Frank, D.N., and Zipris, D. (2012). Prevention of Virus-Induced Type 1 Diabetes with Antibiotic Therapy. J. Immunol. *189*, 3805–3814.

83. Markle, J.G.M., Frank, D.N., Mortin-Toth, S., Robertson, C.E., Feazel, L.M., Rolle-Kampczyk, U., von Bergen, M., McCoy, K.D., Macpherson, A.J., and Danska, J.S. (2013). Sex Differences in the Gut Microbiome Drive

Hormone-Dependent Regulation of Autoimmunity. Science *339*, 1084–1088.

84. Homo Sapiens UCSC Hg19 Human Genome Sequence from iGenome. http://support.illumina.com/sequencing/sequencing_software/igenome.ilmn].

85. Langmead, B., and Salzberg, S.L. (2012). Fast gapped-read alignment with Bowtie 2. Nat. Methods *9*, 357–359.

86. Schloss, P.D., and Westcott, S.L. (2011). Assessing and Improving Methods Used in Operational Taxonomic Unit-Based Approaches for 16S rRNA Gene Sequence Analysis. Appl. Environ. Microbiol. *77*, 3219–3226.

87. Pruesse, E., Quast, C., Knittel, K., Fuchs, B.M., Ludwig, W., Peplies, J., and Glöckner, F.O. (2007). SILVA: a comprehensive online resource for quality checked and aligned ribosomal RNA sequence data compatible with ARB. Nucleic Acids Res. *35*, 7188–7196.

88. Quast, C., Pruesse, E., Yilmaz, P., Gerken, J., Schweer, T., Yarza, P., Peplies, J., and Glöckner, F.O. (2013). The SILVA ribosomal RNA gene database project: improved data processing and web-based tools. Nucleic Acids Res. *41*, D590–D596.

89. Robertson, C.E., Harris, J.K., Wagner, B.D., Granger, D., Browne, K., Tatem, B., Feazel, L.M., Park, K., Pace, N.R., and Frank, D.N. (2013). Explicet: graphical user interface software for metadata-driven management, analysis and visualization of microbiome data. Bioinformatics *29*, 3100–3101.

90. Antonio, M.-B., Y, A.-M.A., I, M.-Z.E., C, C.-M.R., and J, M.-R.M. (2020). Simplified Neutrophil Isolation Protocol. Int. J. Immunol. Immunother. *7*.

STAR★METHODS

KEY RESOURCES TABLE

REAGENT or RESOURCE	SOURCE	IDENTIFIER
Antibodies		
Zombie UV dye	BioLegend	catalog #423108; lot #B440778
BUV395 CD45.2	BD Sciences	clone 104; catalog #564616; lot #3262443; RRID: AB_2738867
BV421 CD11c	BioLegend	clone N418; catalog #117343; lot #B28467; RRID: AB_2563099
BV510 CD64	BioLegend	clone X54-5/7.1; catalog #139335; lot #B407091; RRID: AB_3083124
BV 711 I-A.I-E	BioLegend	clone M5/114.15.2; catalog #107643; lot #B346793; RRID: AB_2565976
PerCP/Cyanine5.5 Ly-6C	BioLegend	clone HK1.4; catalog #128012; lot #B338815; RRID: AB_1659241
PE CD64	BioLegend	clone X54-5/7.1; catalog #139304; lot #B349153; RRID: AB_10612740
APC Ly-6G	BioLegend	clone 1A8; catalog #127614; lot #B296099; RRID: AB_2227348
PE CD45.1	BD Biosciences	clone A20; catalog #553776; lot #3145651; RRID:AB_395044
APC-Cyanine7	BioLegend	clone M1/70; catalog #101226; lot #B445235; RRID: AB_830642
Fc block	BioLegend	catalog #101302; lot #B423718; RRID: AB_312801
Fc Block	BioLegend	catalog #422302; lot#B391743; RRID: AB_2818986
Spark UV 387 CD45	BioLegend	clone HI30; catalog #304085; lot #B398657; RRID: AB_2922537
BV421 CD66b	BioLegend	clone 6/40c; catalog #392915; lot #B428513; RRID: AB_2888722
BV510 HLA-DR	BioLegend	clone L243; catalog #307645; lot #B391691; RRID: AB_2561396
BV711 CD16	BioLegend	clone 3G8; catalog #302043; lot #B371026; RRID: AB_11219184
PerCP/cyanine 5.5 CD24	BioLegend	clone ML5; catalog #311115; lot #B401648; RRID: AB_10962689
PE CD19	BioLegend	clone 4G7; catalog #392506; lot #B369430; RRID: AB_2750097
PE/Dazzle CD11c	BioLegend	clone 3.9; catalog #301641; lot #B391277; RRID: AB_2564082
PE/cyanine 7 CD3	BioLegend	clone OKT3; catalog #317333; lot #B406451; RRID: AB_2561451
APC CD14	BioLegend	clone M5E2; catalog #301807; lot #B372223; RRID: AB_314189
APC-Cyanine7	BioLegend	clone M1/70; catalog #101226; lot #B445235; RRID: AB_830642
anti-neutrophil elastase mAb [NP57]	Abcam	catalog #AB254178; lot #10586222-12,
anti-histone H3 (citrulline R2+R8+R17) antibody	Abcam	catalog #AB5103; lot#1104365-1

(Continued on next page)

Continued		
REAGENT or RESOURCE	SOURCE	IDENTIFIER
goat anti-mouse IgG H&L Alexa Flour 488	Abcam	Catalog # Ab150117
goat anti-rabbit IgG Alexa Fluor 647	Abcam	Catalog # Ab150083
PE-cyanine 7 Anti-Mo TNF alpha	Invitrogen	clone MP6-XT22; catalog #25-7321-82; lot #2250799; RRID: AB_11042728
isotype control IgG1	BioXCell InVivoMAb	clone MOPC-21; catalog #BE0083; lot #722719J2; RRID: AB_1107784
isotype control IgG2A	BioXCell InVivoMAb	clone C1.18; catalog #BE0085; lot#910824J1; RRID: AB_1107771
anti-mouse IFNAR1	BioXCell InVivoMAb	clone MAR1-5A3; catalog #BE0241; lot #829122M1; RRID: AB_2687723
anti-mouse IFNγ	BioXCell InVivoMAb	clone XMG1.2; catalog #BE0055; lot #89072301; RRID: AB_1107694
anti-mouse Ly6G	BioXCell InVivoMAb	clone 1A8; catalog #BE0075-1; lot #854522J3; RRID: AB_1107721
Bacterial and virus strains		
IAV strain x31	Rutigliano et al.[76]	N/A
S. pneumoniae serotype 2 strain D39	Zafar et al.[77]	N/A
S. pneumoniae serotype 7F strain BHN54x	McCullers et al.[78]	N/A
S. pneumoniae serotype 19F strain BHN97	McCullers et al.[78]	N/A
Biological samples		
Patient derived middle ear fluid	University of Colorado Children's hospital	protocol #22-0545
Patient derived blood	University of Colorado Children's hospital	protocol #22-0545
Patient derived nasal and nasopharyngeal swabs	University of Colorado Children's hospital	protocol #22-0545
Healthy human blood	University of Colorado Anschutz	protocols #05–0993; #22-0545
Chemicals, peptides, and recombinant proteins		
Human recombinant IFNα2	Peprotech	catalog #300-02AA-100UG; lot #03211688
Human recombinant IFNβ	Peprotech	catalog #300-02BC-50UG; lot #0316S458-1
SYTOX green	Invitrogen	catalog #S7020; lot #2901546
GolgiStop™ protein transport inhibitor	BD biosciences	catalog #555029; lot #0227731
DAPI	Roche	catalog #10236276001; lot #68732323
Mouse recombinant IFNα2	R&D Systems	catalog# 10149-IF
Mouse recombinant IFNβ	R&D Systems	catalog# 8234-MB
Critical commercial assays		
Legendplex Human Anti-virus Response Panel 1	BioLegend	catalog #741270; lot #B485166
Legendplex Human Essential Immune Response Panel	BioLegend	catalog #740930
Legendplex Mouse Anti-Virus Response Panel	BioLegend	catalog #740622
BD sciences Mouse IFNγ ELISA set	BD sciences	catalog #555138; lot #3243313; RRID: AB_2869028
BD sciences Mouse TNF ELISA set	BD sciences	catalog #555268; lot #2189147; RRID: AB_2869055
DuoSet Mouse CXCL2/MIP-2	R&D systems	catalog #DY452-05; lot #P364430
DuoSet Mouse Myeloperoxidase	R&D systems	catalog #DY3667; lot #P364491
Experimental models: Organisms/strains		
C57BL/6J mice	The Jackson Laboratory	catalog #000664, RRID:IMSR_JAX:000664
Ifnar$^{-/-}$ mouse strain B6J.129S2-*Ifnar1*^{tm1Agt}/Mmjax	The Jackson Laboratory	RRID:MMRRC_032045-JAX
LysM^{cre} (B6.129P2-*Lyz2*$^{tm1(cre)Ifo}$/J)	The Jackson Laboratory	catalog #004781, RRID:IMSR_JAX:004781
IFNAR^{flox} B6(Cg)-*Ifnar1*$^{tm1.1Ees}$/J	The Jackson Laboratory	catalog #028256, RRID:IMSR_JAX:028256

(*Continued on next page*)

CellPress
OPEN ACCESS

Continued

REAGENT or RESOURCE	SOURCE	IDENTIFIER
Software and algorithms		
LEGENDplex™ Data Analysis Software Suite	BioLegend	https://legendplex.qognit.com/user/login?next=workflow.load_data&assay_id=87667
Prism	GraphPad, version 10	RRID:SCR_002798
NIS-Elements AR 4.60.00 64-bit software	Nikon Elements	RRID:SCR_014329
FlowJo™ Software, version 10.1	BD Life Sciences	RRID:SCR_008520
Explicet	Explicet	v2.10.5, www.explicet.org
Deposited data		
16S rRNA sequencing data	NCBI Short Read Archive	BioProject: PRJNA1404333
Other		
LSR Fortessa X-20	University of Colorado Flow Cytometry Shared Resource	RRID:SCR_022035
Cytek Aurora 5 color spectral analyzer	University of Colorado Flow Cytometry Shared Resource	RRID:SCR_022035
Synergy HTX plate reader	BioTek	RRID:SCR_020536

EXPERIMENTAL MODEL AND STUDY PARTICIPANT DETAILS

Human subjects

Clinical samples were collected from male and female children with a clinical diagnosis of rAOM or COME under Institutional Review Board approval (Table 1). Samples included nasal swabs, nasopharyngeal swabs, venipuncture for blood collection, and middle ear fluid. Nasal and nasopharyngeal swabs were frozen at −80°C. Blood samples were processed immediately for neutrophil isolation (see below). Middle ear fluid samples were collected from children undergoing surgery for the placement of tympanostomy tubes, with physician characterization of effusions as purulent, serous, or mucoid based on appearance. Middle ear fluid was either directly frozen at −80°C or processed immediately for immune cell analysis by flow cytometry. Sample sizes for all analyses are indicated in Figure legends. The influence of sex, race and ethnicity on study results was not analyzed, as the study was underpowered to address these factors. All work with human samples was approved by the University of Colorado Institutional Review Board (protocols #05–0993 and #22–0545) on samples from consented participants.

Animals

Adult male and female mice aged 8–12 weeks were used for these studies. C57BL/6J (WT) mice were purchased from The Jackson Laboratory (#000664). The *Ifnar*$^{-/-}$ mouse strain B6J.129S2-*Ifnar1*^{tm1Agt}/Mmjax, RRID:MMRRC_032045-JAX was obtained from the Mutant Mouse Resource and Research Center (MMRRC) at The Jackson Laboratory, an NIH-funded strain repository, and was donated to the MMRRC by Michel Aguet, Ph.D., Swiss Institute for Experimental Cancer Research. LysM^{cre} x IFNAR$^{fl/fl}$ mice were created by crossing LysM^{cre} (B6.129P2-*Lyz2*$^{tm1(cre)Ifo}$/J, The Jackson Laboratory #004781) and IFNAR^{flox} (B6(Cg)-*Ifnar1*$^{tm1.1Ees}$/J, The Jackson Laboratory #028256) as previously described, with genotype confirmation by polymerase chain reaction (PCR).[41,42] All mouse strains used in these studies are on the C57BL/6J genetic background. Mice were maintained in the University of Colorado Office of Laboratory Animal Resources. Housing conditions included a light cycle of 14:10 (light:dark) hours, a temperature of 72 ± 2°F, with 40 ± 10% humidity. Animals were housed in groups of 5 or fewer individuals per cage and littermates were randomly assigned to experimental groups. Studies were underpowered for analysis of the impact of sex on study results, and as a result this analysis was not performed. All animal studies were approved by the Animal Care and Use Committee of the University of Colorado School of Medicine (protocol #00927).

Bacteria

S. pneumoniae strains used in this study included a streptomycin resistant variant of serotype 2 strain D39,[79] serotype 7F strain BHN54x,[80] and serotype 19F strain BHN97.[80] *S. pneumoniae* was grown on Tryptic Soy agar plates containing neomycin (5 μg/mL, Sigma) and streptomycin (only for serotype 2, 50 μg/mL) with fresh catalase (5,000 units/plate, Worthington Biomedical Corporation). Growth from overnight plates or glycerol stocks were inoculated into Todd Hewitt Broth with 5% Yeast Extract (BD Bacto), with 50 μg/mL streptomycin (Sigma), added for type 2 strain D39 only, at 37°C with 5% CO_2. All studies with *S. pneumoniae* were approved by the University of Colorado Institutional Biosafety Committee (protocol #1418).

Viruses

Stocks of IAV strain x31, which contains the internal genes of PR8 (A/Puerto Rico/8/34) with the H3N2 surface proteins from A/Hong Kong/1/1968,[81] were kindly provided by Dr. Jenna Guthmiller, University of Colorado Anschutz Medical School. All studies with IAV were approved by the University of Colorado Institutional Biosafety Committee (protocol #1418).

METHOD DETAILS

High-throughput DNA sequencing for microbiome analysis

16S amplicon library construction

Bacterial profiles were determined by broad-range amplification and sequence analysis of 16S rRNA genes following our previously described methods.[82,83] In brief, amplicons were generated using primers that target approximately 300 base pairs of the V1V2 variable region of the 16S rRNA gene. PCR products were normalized using agarose gel densitometry, pooled, lyophilized, purified and concentrated using a DNA Clean and Concentrator Kit (Zymo, Irvine, CA). Pooled amplicons were quantified using Qubit Fluorometer 2.0 (Invitrogen, Carlsbad, CA). The pool was diluted to 4 nM and denatured with 0.2 N NaOH at room temperature. The denatured DNA was diluted to 15 pM and spiked with 25% of the Illumina PhiX control DNA prior to loading the sequencer. Illumina paired-end sequencing was performed on the Miseq platform using a 500 cycle version 2 reagent kit.

Analysis of Illumina paired-end reads

Illumina Miseq paired-end reads were aligned to human reference genome Hg19 with bowtie2 and matching sequences discarded.[84,85] As previously described, the remaining non-human paired-end sequences were sorted by sample via barcodes in the paired reads with a python script.[83] Sorted paired end sequence data were deposited in the NCBI Short Read Archive under BioProject: PRJNA1404333. The sorted paired reads were assembled using phrap.[77,78]. Pairs that did not assemble were discarded. Assembled sequence ends were trimmed over a moving window of 5 nucleotides until average quality met or exceeded 20. Trimmed sequences with more than 1 ambiguity or shorter than 200 nt were discarded. Potential chimeras identified with Uchime (usearch6.0.203_i86linux32)[76] using the Schloss[86] Silva reference sequences were removed from subsequent analyses. Assembled sequences were aligned and classified with SINA (1.3.0-r23838)[87] using the 418,497 bacterial sequences in Silva 115NR99[88] as reference configured to yield the Silva taxonomy. Operational taxonomic units (OTUs) were produced by clustering sequences with identical taxonomic assignments. This process generated 903,141 sequences for 29 samples (average sequence length: 315 nt; average sample size: 31,142 sequences/sample; minimum sample size: 5,210; maximum sample size: 100,901). The median Goods coverage score was ≥99.5% at the rarefaction point of 5,210. The software package Explicet (v2.10.5, www.explicet.org)[89] was used for display, analysis (rarefied values for median Good's coverage), and figure generation of results.

Human neutrophil isolation

Human neutrophils were isolated from subjects who consented to participation under Institutional Review Board approval. Following 30 mL venipuncture blood collection into tubes containing acid citrate/dextrose, neutrophils were purified as previously described.[90] Briefly, 10 mL aliquots were placed into conical tubes with 10 mL of 3% dextran in PBS and mixed gently at room temperature. After red blood cells (RBCs) agglutination, the top layer of plasma was removed and combined with PBS to a total volume of 50 mL. Samples were centrifuged at 300 xg for 15 min, decanted, and pellets resuspended in 30 mL of PBS. PBS resuspension was overlayed onto 15 mL of Histopaque 1077 (Sigma) and centrifuged at 750 xg for 25 min (without break). Remaining RBCs were lysed from cell pellets, cells were washed twice with PBS for 10 min at 300 xg and isolated neutrophils were counted using trypan blue (0.4% in PBS) and a hemacytometer (Hausser scientific) on a motic AE2000 microscope.

Mouse infection

S. pneumoniae infections were preformed using serotypes 2, 7F, and 19F. *S. pneumoniae* inoculum was prepared from frozen aliquots, following centrifugation at ≥10,000 xg and resuspension in PBS for intranasal injection in a total volume of 50 μL, with mice anesthetized with inhaled isoflurane. For poly(I:C) co-treatment, mice were intranasally injected with 50 μL of 1 mg/mL of poly I:C (InVivoGen) or 50 μL sterile PBS as a vehicle control, with a 5 min rest between *S. pneumoniae* infection and poly(I:C) injection. For IAV infections, frozen aliquots of live IAV x31 were diluted in PBS and intranasally injected in a total volume of 25 μL. Mice were infected with IAV 72 h prior to *S. pneumoniae* challenge. Control lung infections were conducted via intratracheal injection of *S. pneumoniae*. For recombinant IFN treatments, mice were intranasally injected with 50 μL of 1 μg each of recombinant IFNα2 (R&D Systems, # 10149-IF) and IFNβ (R&D Systems, # 8234-MB), or 50 μL of PBS as a vehicle control. For cell and cytokine depletions, mice were treated intraperitoneally (i.p.) 24 h prior to *S. pneumoniae* infection with 200 μg/mouse with isotype control IgG1 (BioXCell InVivoMAb clone MOPC-21, catalog #BE0083, lot #722719J2), isotype control IgG2A (BioXCell InVivoMAb clone C1.18, catalog #BE0085, lot#910824J1), anti-mouse IFNAR1 (BioXCell InVivoMAb clone MAR1-5A3, catalog #BE0241, lot #829122M1), anti-mouse IFNγ (BioXCell InVivoMAb clone XMG1.2, catalog #BE0055, lot #89072301), or anti-mouse Ly6G (BioXCell InVivoMAb clone 1A8, catalog #BE0075-1, lot #854522J3).

At study endpoints, nasal lavages were collected and middle ear cavities were surgically removed. Serum was collected following cardiac puncture. Nasal lavages were performed following tracheal incision and flushing of the nasopharynx with PBS, collected through the nares. Nasopharyngeal burdens were enumerated following serial dilution on Tryptic Soy agar plates containing

neomycin (5 µg/mL, Sigma) and streptomycin (only for serotype 2, 50 µg/mL) with fresh catalase (5,000 units/plate, Worthington Biomedical Corporation). For middle ear CFU enumeration, samples were homogenized in a Bullet Blender tissue homogenizer (Stellar Scientific) in PBS prior to serial dilution. Bacterial burdens were enumerated following incubation of agar plates at 37°C with 5% CO2 for 18–24 h.

Bone marrow chimeras

For the generation of bone marrow chimeras, CD42.1^{+} WT mice received two doses of 500 rads 90 min apart. Bone marrow transfers were conducted 24 h following irradiation, with each mice receiving 3x10^{6} cells purified from CD45.2^{+} WT or *Ifnar*$^{-/-}$ donors of matched sex, with cells delivered via tail vein injection in 200 µL. Mice were allowed 5–8 weeks for reconstitution prior to challenge with 10^{5} PFU of x31 IAV or PBS control delivered intranasally, followed by intranasal infection with *S. pneumoniae* serotype 2 or 7F at 72 h post-IAV. Nasal lavage and middle ear samples were collected at 24 h post-infection with *S. pneumoniae* for processing for CFU enumeration and flow cytometry, as above. Reconstitution of the hematopoietic system with donor-derived cells was >80% in all mice as measured by CD45.1/2 staining.

Flow cytometry

Mouse middle ear samples collected for immune cell analysis by flow cytometry were subjected to gentle homogenization with enzymatic digestion using DNAseI (30 µg/mL, Sigma), type 4 collagenase (1 mg/mL, Worthington Biochemical Corporation) in HBSS(+) (with calcium and magnesium, Gibco). After digestion, samples filtered through a 70 µM strainer and RBCs were lysed using RBC lysis buffer (0.15M NH_4Cl, 10mM $KHC0_3$, 0.1mM Na_2EDTA, pH 7.4). Live/Dead staining was performed using Zombie UV dye (BioLegend, #423108, lot #B440778) in PBS at 4°C for 20 min. Following live/dead staining cells were washed in FACs buffer (1% BSA, 0.01% NaN_3, PBS) centrifuged at 500 xg and resuspended in Fc block (BioLegend, catalog #101302, lot #B423718 for mouse samples or catalog #422302 lot#B391743 for human cells) and incubated at 4°C for 20 min. For surface staining, cells were incubated for 30 min at 4°C in FACS buffer with the following anti-mouse antibodies: BUV395 CD45.2 (BD biosciences, clone 104, catalog #564616, lot #3262443), BV421 CD11c (BioLegend, clone N418, catalog #117343, lot #B284676), BV510 CD64 (BioLegend, clone X54-5/7.1, catalog #139335, lot #B407091), BV 711 I-A.I-E (BioLegend, clone M5/114.15.2, catalog #107643, lot #B346793), PerCP/Cyanine5.5 Ly-6C (BioLegend, clone HK1.4, catalog #128012, lot #B338815), PE CD64 (BioLegend, clone X54-5/7.1, catalog #139304, lot #B349153), APC Ly-6G (BioLegend, clone 1A8, catalog #127614, lot #B296099), PE CD45.1 (BD, clone A20, catalog #553776, lot #3145651), APC-Cyanine7 (BioLegend, clone M1/70, catalog #101226, lo t#B445235). Anti-human antibodies included: Spark UV 387 CD45 (BioLegend, clone HI30, catalog #304085, lot #B398657), BV421 CD66b (BioLegend, clone 6/40c, catalog #392915, lot #B428513), BV510 HLA-DR (BioLegend, clone L243, catalog #307645, lot #B391691), BV711 CD16 (BioLegend, clone 3G8, catalog #302043, lot #B371026), PerCP/cyanine 5.5 CD24 (BioLegend, clone ML5, catalog #311115, lot #B401648), PE CD19 (BioLegend, clone 4G7, catalog #392506, lot #B369430), PE/Dazzle CD11c (BioLegend, clone 3.9, catalog #301641, lot #B391277), PE/cyanine 7 CD3 (BioLegend, clone OKT3, catalog #317333, lot #B406451), APC CD14 (BioLegend, clone M5E2, catalog #301807, lot #B372223), APC-Cyanine7 (BioLegend, clone M1/70, catalog #101226, lot #B445235). After staining and washing, cells were fixed in 1% paraformaldehyde in PBS prior to analysis on a Cytek Aurora 5 color spectral analyzer in the University of Colorado Flow Cytometry Shared Resource (RRID:SCR_022035). Data analysis was performed using FlowJo Software, version 10.1 (BD Life Sciences).

For intracellular flow cytometry, cells were incubated with GolgiStop protein transport inhibitor (BD biosciences, #555029, lot #0227731) in RP10 media (RPMI 1640 with L-glutamine and 10% FBS) and incubated at 37°C with 5% CO2 for 3 h prior to staining. After surface staining, cells were washed in FACs buffer and incubated in PFA-saponin at room temperature for 15 min. Cells were centrifuged at 600 xg for 7 min at 4°C, and washed twice with PBS-Saponin prior to intracellular staining. For intracellular staining, cells were incubated for 45 min at room temperature in PBS-saponin buffer with PE-cyanine 7 Anti-Mo TNF alpha (Invitrogen, clone MP6-XT22, catalog #25-7321-82, lot #2250799). After staining, cells were washed twice in PBS-saponin and resuspended in FACS buffer prior to spectral analysis.

For detection of intracellular ROS, cells were incubated prior to antibody staining with the ROS probe Dihydrorhodamine (DHR) 123 (Sigma) in FACs buffer for 30 min at 37°C with 5% CO_2. After probe incubation, cells were washed and staining proceeded as above.

Neutrophil functional assays

For opsonophagocytic assays, FITC-labeled aliquots of heat-killed (HK) *S. pneumoniae* serotype 2 were prepared. Bacteria were killed by incubation at 65°C for 1 h, with killing confirmed by the absence of growth on agar plates. HK bacteria were incubated with 0.5 mg/mL FITC diluted in PBS for 1 h at 4°C, followed by two washes and final resuspension in HBSS(−), (no calcium or magnesium, Gibco) + 1% bovine serum albumin (BSA). 2×10^{6} CFU of HK FITC-*S. pneumoniae* was pre-opsonized in 7.6% baby rabbit serum (MP biomedicals) for 30 min at 37°C under rotation. Bacteria were incubated with isolated single cell suspensions (prepared as for flow cytometry) or purified human neutrophils in HBSS(−) + 1% BSA for 30 min at 37°C under rotation prior to antibody staining as above. To assess the percentage of intracellular bacteria detected in the opsonophagocytic assay, samples were incubated with or without 0.25 mg/mL trypan blue (Thermo Scientific Chemicals) to quench extracellular FITC for 5 min at 4°C. Cells were washed and resuspended prior to antibody staining.

For analysis of NETosis by SYTOX green staining, purified neutrophils were incubated at a density of 50,000 cells in RP10 media with or without 1 μg/well recombinant IFNα2 and 1 μg/well IFNβ (Peprotech, catalog #300-02AA-100UG, #300-02BC-50UG), 10 μM phorbol myristate acetate (PMA), (Sigma), or in 0.1% Triton X-100 detergent for 3 h at 37°C with 5% CO_2. Following incubation, SYTOX green (Invitrogen, catalog #S7020) was added at a concentration of 1 μM per well and incubated in light protected conditions for 15 min at room temperature. Fluorescence was measured at 485 nm excitation, 527 nm emission using a Synergy HTX plate reader (BioTek). RFUs were calculated relative to values in untreated wells.

Cytokine and chemokine analysis

Clinical middle ear fluid samples were centrifuged for 10 min at 500 xg for cytokine/chemokine analysis. Middle ear samples from mice were homogenized prior to centrifugation for 10 min at 500 xg. Cytokines and chemokines were detected in supernatants using a bead-based flow cytometry multiplex assay (LEGENDplex, BioLegend), with samples detected on an LSR Fortessa X-20 in the the University of Colorado Flow Cytometry Shared Resource (RRID:SCR_022035). CXCL2 was analyzed using CXCL2/MIP-2 ELISA kits (R&D Systems), and MPO was detected using an MPO ELISA kit (R&D systems). Additional samples were analyzed for TNFα and IFNγ using ELISA kits (BD). Data were analyzed using the LEGENDplex Data Analysis Software Suite (BioLegend) and in Prism (GraphPad, version 10).

Histology

For analysis of NETosis by histology, 50,000 human neutrophils were incubated on coverslips in RP10 with or without 1 μg/well each recombinant IFNα2 and IFNβ, as above, for 3 h at 37°C with 5% CO_2. Cells were then fixed onto cover slips with 4% paraformaldehyde in PBS for 20 min at room temperature. Coverslips were washed twice with PBS and blocked with 1% BSA, 5% normal goat serum (Invitrogen) in PBS for 1 h at 4°C and washed twice with PBS prior to staining. Cells were stained with 1:250 of anti-neutrophil elastase mAb [NP57] (Abcam, catalog #AB254178, lot #10586222-12) and anti-histone H3 (citrulline R2+R8+R17) antibody (Abcam, catalog #AB5103, lot#1104365-1) in blocking buffer overnight at 4°C. Following overnight incubation coverslips washed twice in PBS and a secondary stain was applied with goat anti-mouse IgG H&L Alexa Flour 488 (Abcam, Ab150117) and goat anti-rabbit IgG Alexa Fluor 647 (Abcam, Ab150083) in PBS for 1 h at room temperature while light protected. Coverslips were washed twice with PBS and then stained with DAPI at 1:1000 (Roche, catalog #10236276001) for 5 min at room temperature and washed twice before applying ProLong glass antifade mountant (Invitrogen) to microscope slides (VWR). Mounting media was solidified for 36 h at 4°C and imaged on a Nikon eclipse Ti-S microscope. Images were analyzed with NIS-Elements AR 4.60.00 64-bit software by investigators blinded to study groups.

QUANTIFICATION AND STATISTICAL ANALYSIS

Statistical analysis was conducted using Prism (GraphPad Software; version 10). Data with normalized distribution (Shapiro-Wilk test) were analyzed using two-tailed Student's *t* tests or analysis of variance (ANOVA) tests with Dunnett's, Sidak's, or Tukey's post hoc analysis for multiple comparisons, as specified. Data with non-Gaussian distributions were analyzed using two-tailed Mann-Whitney U tests or Kruskal-Wallis tests with Dunn's post hoc analysis for multiple comparisons. Correlations were assessed using Pearson correlation coefficient. Significant outliers were detected using the ROUT method (Q = 1%) and removed. p values of <0 .05 were considered significant. Statistical tests, the definition and value of n, and dispersion and precision measures for each experiment are indicated in Figure Legends.

Cell Reports Medicine, Volume *7*

Supplemental information

Type I interferon signaling in hematopoietic cells impairs neutrophil antibacterial function in the middle ear during viral co-infection

Steven C. Shaw, Taylor L. Jamil, Gabriela Heslop, Jeremy T. Fleck, Wyatt Johnson, Brian P. Lorenz, Zoe Drigot, J. Kirk Harris, Sarah A. Gitomer, and Sarah E. Clark

Supplementary Information

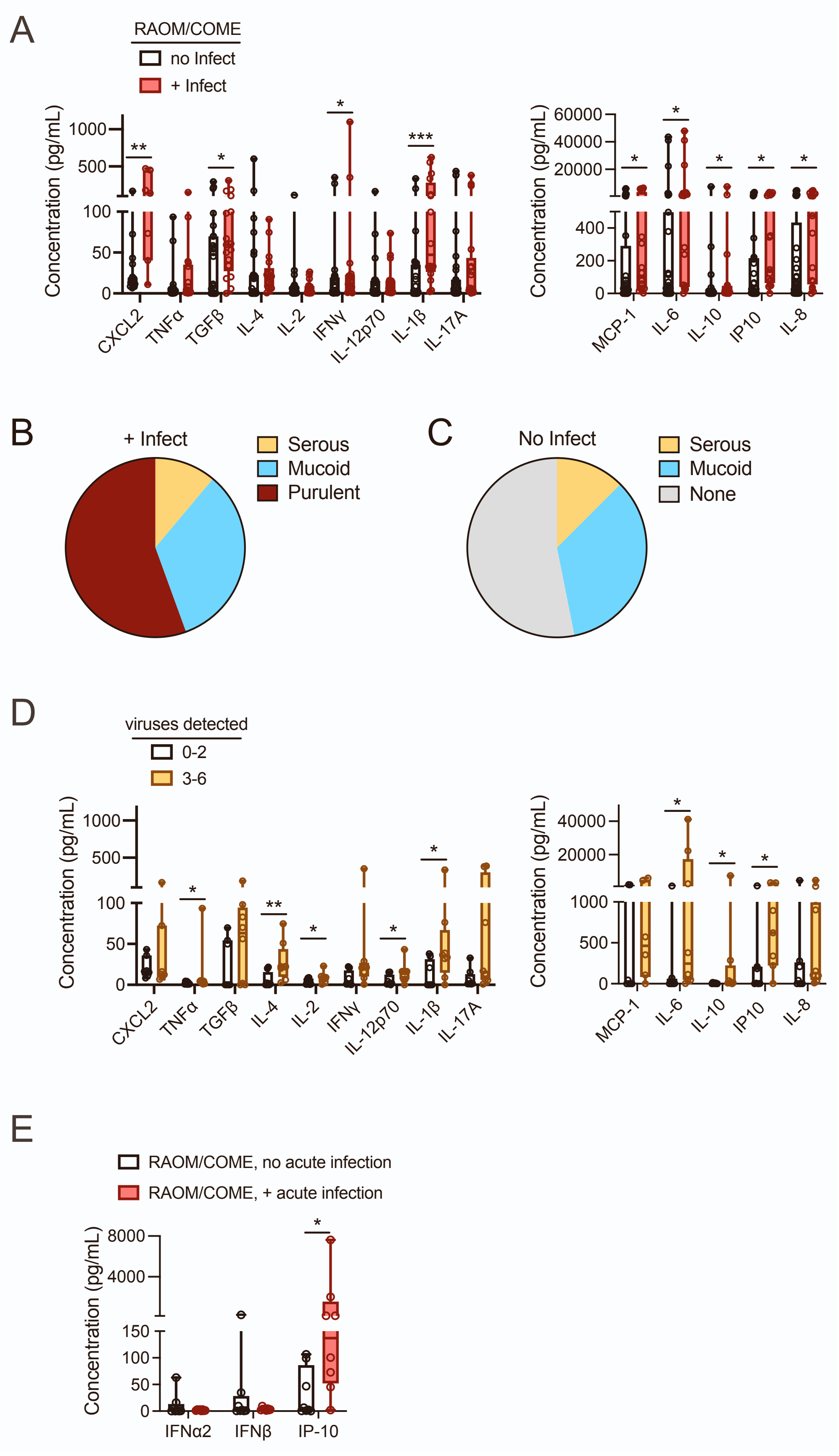
A
RAOM/COME
no Infect
+ Infect
Concentration (pg/mL)
CXCL2
TNFα
TGFβ
IL-4
IL-2
IFNγ
IL-12p70
IL-1β
IL-17A
MCP-1
IL-6
IL-10
IP10
IL-8
B
+ Infect
Serous
Mucoid
Purulent
C
No Infect
Serous
Mucoid
None
D
viruses detected
0-2
3-6
E
RAOM/COME, no acute infection
RAOM/COME, + acute infection
IFNα2
IFNβ
IP-10

Supplementary Figure 1 (related to Figure 1). Middle ear effusion characteristics. (A) Concentration of inflammatory cytokines and chemokines detected in middle ear fluid samples from children with rAOM or COME with a clinical diagnosis of acute OM (+ Infect, n=18 subjects) or no diagnosis of acute OM (no Infect, n=31 subjects), individual values plotted from Figure 1C. (B-C) Percentage of patient samples with middle ear effusions detected, with effusions classified as serous, mucoid, or purulent, among children with rAOM or COME with a clinical diagnosis of active OM (+ Infect, n=18 subjects), (B) or no clinical diagnosis of active OM (No Infect, n=31 subjects), (C). (D) Concentration of inflammatory cytokines and chemokines detected in middle ear fluid samples from children with rAOM or COME with 0-2 individual viruses detected (n=8 subjects) or 3-6 viruses detected (n=8 subjects), from Figure 1D. (E) Concentration of IFNα2, IFNβ, and IP-10 (CXCL10) detected in middle ear fluid samples from children with rAOM or COME with a clinical diagnosis of acute OM (+ Infect, n=8 subjects) or no diagnosis of acute OM (no Infect, n=8 subjects), detected in the subset of available samples from (A). Samples at or below the limit of detection are reported as 0 pg/mL. Box boundaries indicate the 25th and 75th percentiles, with a horizontal line representing the median and whiskers indicating minimum and maximum values. *p<.05, **p<.01, ***p<.001, Mann-Whitney U test.

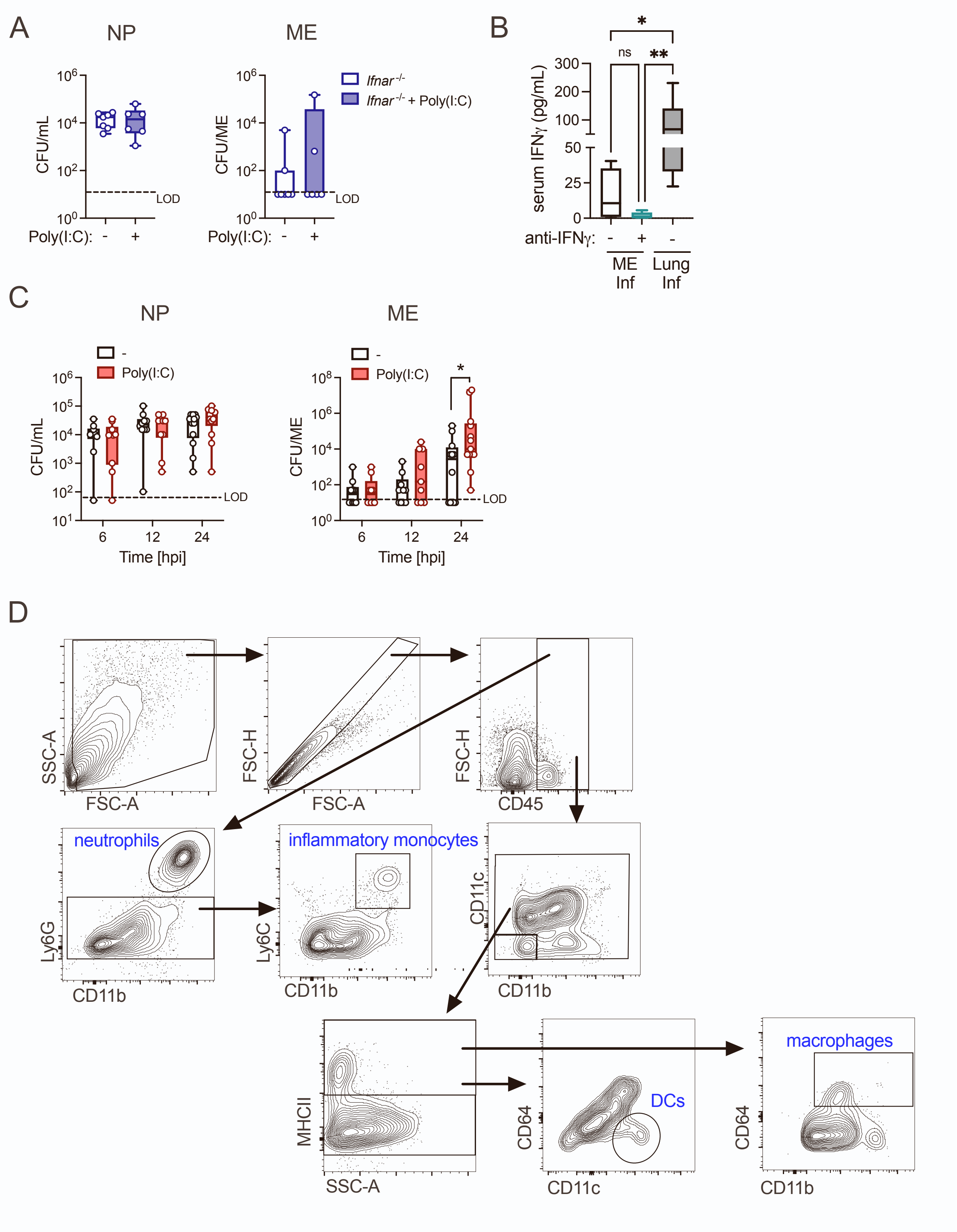

Supplementary Figure 2 (related to Figures 3-4). IFNγ depletion and middle ear infection time course. (A) Burden of *S. pneumoniae* serotype 7F detected in the nasopharynx (NP) and middle ear (ME) of *Ifnar*$^{-/-}$ mice at 24

hours post-infection (10^5 CFU/mouse i.n.) with or without co-treatment with poly(I:C) (50 μg/mouse i.n.), n=6-7 mice/grp. (B) Concentration of IFNγ detected in the serum of WT mice infected with *S. pneumoniae* intranasally (10^5 CFU/mouse i.n.) to induce middle ear (ME) infection or intratracheally ($5x10^6$ CFU/mouse i.t.) to induce lung infection 24 hours following treatment with anti-IFNγ antibody or isotype control antibody (200 μg/mL i.p.), n=7-9 mice/grp. (C) Burden of *S. pneumoniae* detected at indicated time points post-infection (10^5 CFU/mouse i.n.) with or without co-treatment with poly(I:C), n= 10 mice/grp (6 h, 12 h), n= 19 mice/grp (24 h). (D) Gating strategy to detect neutrophils ($CD45^+Ly6G^+CD11b^+$), inflammatory monocytes ($CD45^+Ly6G^-Ly6C^+CD11b^+$), dendritic cells (DCs, $CD45^+MHCII^+CD64^-CD11c^+$), and macrophages ($CD45^+CD64^+CD11b^+$) in middle ear samples by flow cytometry. Data pooled from 2 (A) or 3 (B, C) independent experiments. Box boundaries indicate the 25th and 75th percentiles, with a horizontal line representing the median and whiskers indicating minimum and maximum values. LOD = limit of detection. *p<.05, **p<.01, one-way ANOVA with Tukey's post hoc test (B), Mann-Whitney U test (C).

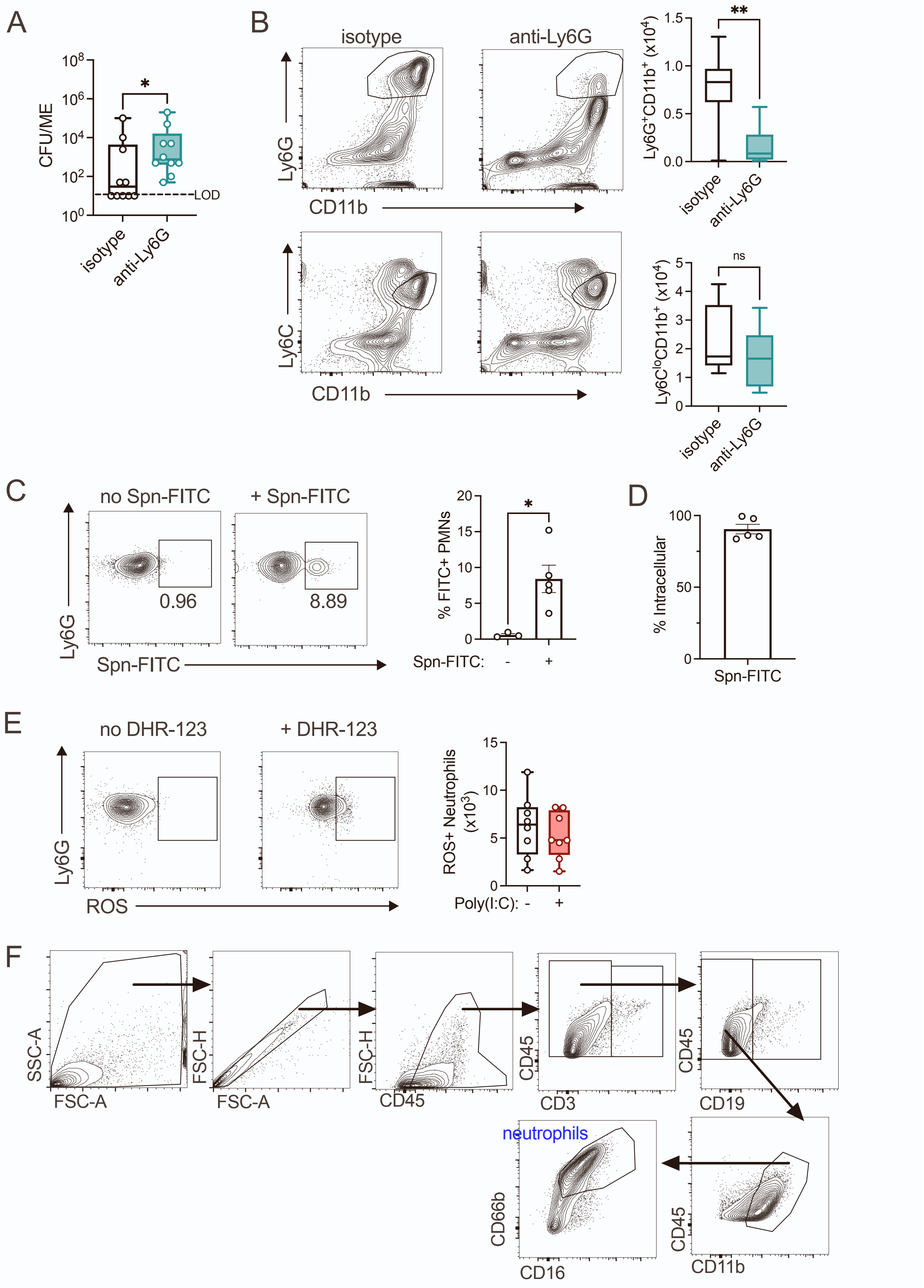

A
CFU/ME
10^8
10^6
10^4
10^2
10^0
*
LOD
isotype
anti-Ly6G
B
isotype
anti-Ly6G
Ly6G
CD11b
Ly6C
Ly6G+CD11b+ (x10^4)
**
ns
Ly6C^loCD11b+ (x10^4)
C
no Spn-FITC
+ Spn-FITC
0.96
8.89
Spn-FITC
% FITC+ PMNs
Spn-FITC: - +
D
% Intracellular
E
no DHR-123
+ DHR-123
ROS
ROS+ Neutrophils (x10^3)
Poly(I:C): - +
F
SSC-A
FSC-A
FSC-H
CD45
CD3
CD19
neutrophils
CD66b
CD16
CD11b

Supplementary Figure 3 (related to Figure 5). Neutrophil depletion and detection of neutrophil phagocytosis and ROS. (A) Burden of *S. pneumoniae* serotype 7F detected in the middle ear of WT mice at 24 hours post-infection (10^5 CFU/mouse i.n.) in mice treated 24 hours prior to infection with anti-Ly6G antibody or isotype control antibody (200 µg/mL i.p.), n=10 mice/grp. (B) Representative flow cytometry plots and total number of $Ly6G^+CD11b^+$ gated neutrophils and $Ly6C^{lo}CD11b^+$ gated neutrophils in mice from (A). (C) Representative flow cytometry plots and percentage of Spn-$FITC^+$ neutrophils detected following 1 hour incubation with or without FITC-labeled heat-killed *S. pneumoniae*, for neutrophils detected in the blood of WT naïve mice, n=3-5 mice/grp. (D) Percentage of intracellular *S. pneumoniae* detected by Spn-FITC uptake assay. (E) Representative flow cytometry plots and total number of ROS+ neutrophils detected with the DHR-123 probe by flow cytometry in the middle ear of WT mice at 24 hours post-infection with *S. pneumoniae* (10^5 CFU/mouse i.n.) with or without co-treatment with poly(I:C) (50 µg/mouse i.n.), n=8 mice/grp. (F) Gating strategy to detect human neutrophils ($CD45^+CD3^-CD19^-CD11b^+CD66b^+CD16^+$) from blood and middle ear samples, with representative plots from a blood sample. Data pooled from 2 (C, D) or 3 (A, B, E) independent experiments. Box boundaries indicate the 25th and 75th percentiles, with a horizontal line representing the median and whiskers indicating minimum and maximum values. LOD = limit of detection. *p<.05, Mann-Whitney U test (A, B), unpaired t test (C).

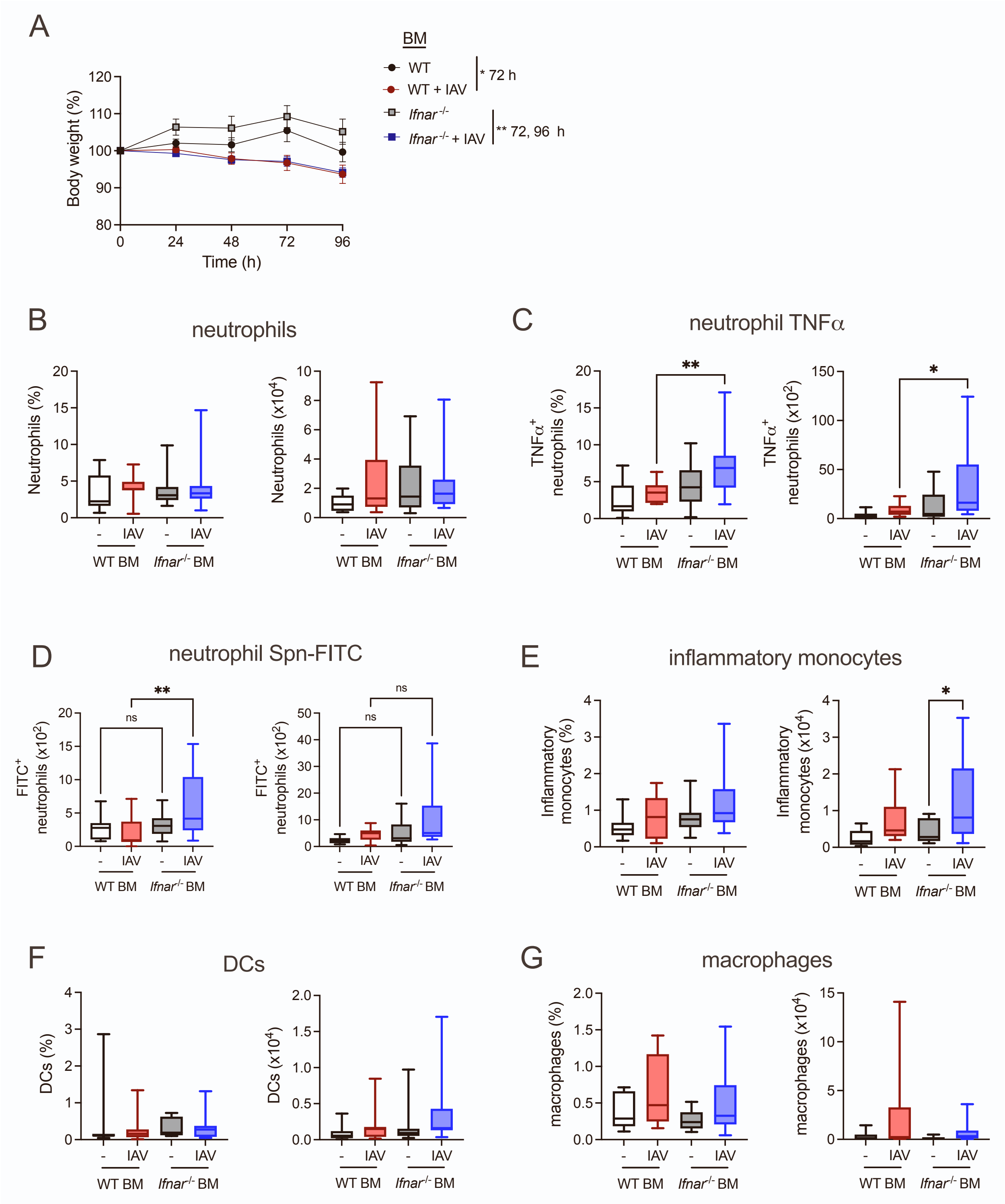

Supplementary Figure 4 (related to Figure 6). Impact of IFNAR signaling in irradiation-sensitive cells on immune cell recruitment and activation in the middle ear during *S. pneumoniae* serotype 2 infection. (A) Percent of starting body weight over time in irradiated WT recipients of WT or *Ifnar*$^{-/-}$ bone marrow with or without IAV co-infection (10^5 PFU/mouse i.n.) 72 hours prior to challenge with *S. pneumoniae* serotype 2 (10^7 CFU/mouse i.n.), n=10-13 mice/grp, plotted as mean +/- SEM. (B-G) Percent and total number of neutrophils (B), TNFα$^+$ neutrophils (G), Spn-FITC$^+$ neutrophils, detected following 1 hour incubation with or without FITC-labeled heat-

killed *S. pneumoniae*, (D), inflammatory monocytes (E), DCs (F), and macrophages (G) detected by flow cytometry in the middle ear of bone marrow chimeric mice from (A). Data pooled from 3 independent experiments. Box boundaries indicate the 25th and 75th percentiles, with a horizontal line representing the median and whiskers indicating minimum and maximum values. *p<.05, **p<.01, Welch's t test (A), one-way ANOVA with Sidak's post hoc test (B-G).

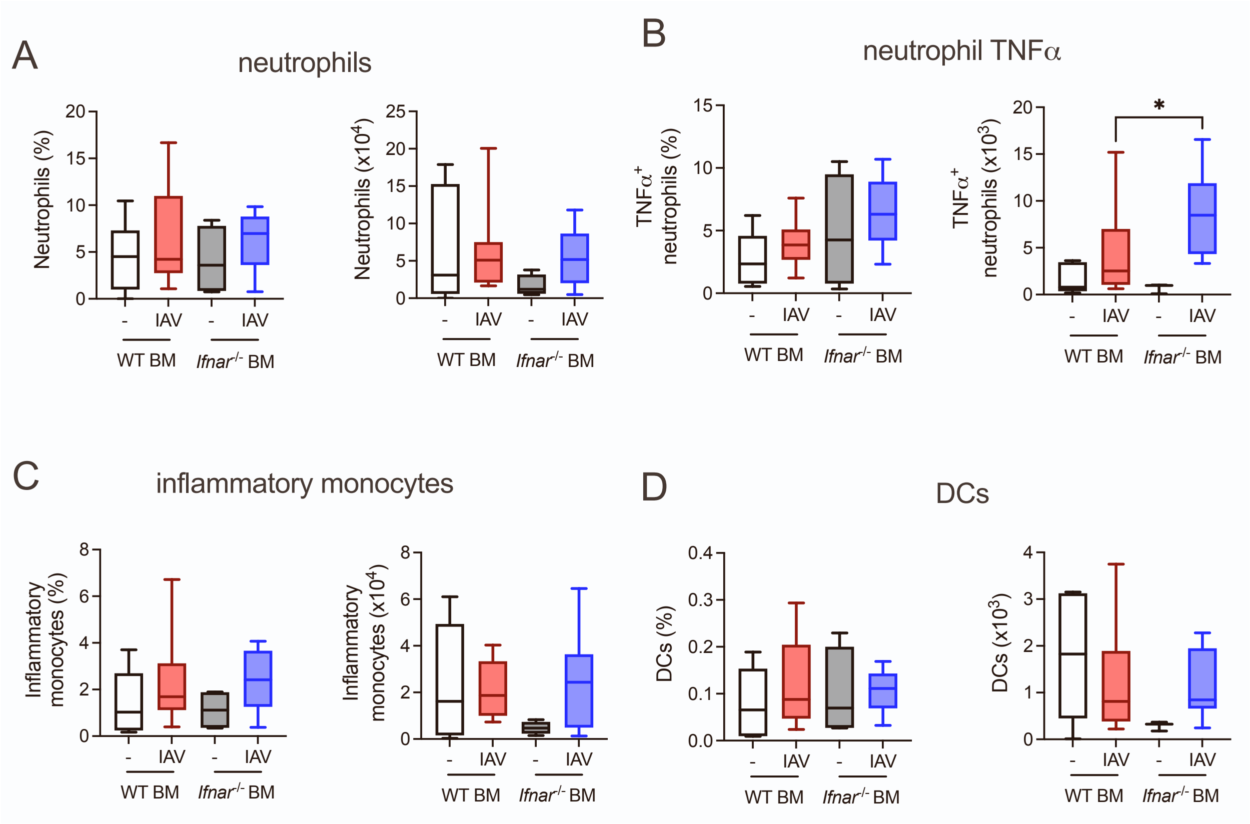

Supplementary Figure 5 (related to Figure 6). Impact of IFNAR signaling in irradiation-sensitive cells on immune cell recruitment and activation in the middle ear during *S. pneumoniae* serotype 7F infection. (A-D) Percent and total number of neutrophils (A), TNFα$^+$ neutrophils (B), inflammatory monocytes (C), and DCs (D) detected by flow cytometry in the middle ear of irradiated WT recipients of WT or *Ifnar*$^{-/-}$ bone marrow with or without IAV co-infection (10^5 PFU/mouse i.n.) 72 hours prior to challenge with *S. pneumoniae* serotype 7F (10^5 CFU/mouse i.n.), n=7-10 mice/grp. Data pooled from 3 independent experiments. Box boundaries indicate the 25th and 75th percentiles, with a horizontal line representing the median and whiskers indicating minimum and maximum values. **p*<.05, one-way ANOVA with Sidak's post hoc test (B-G).

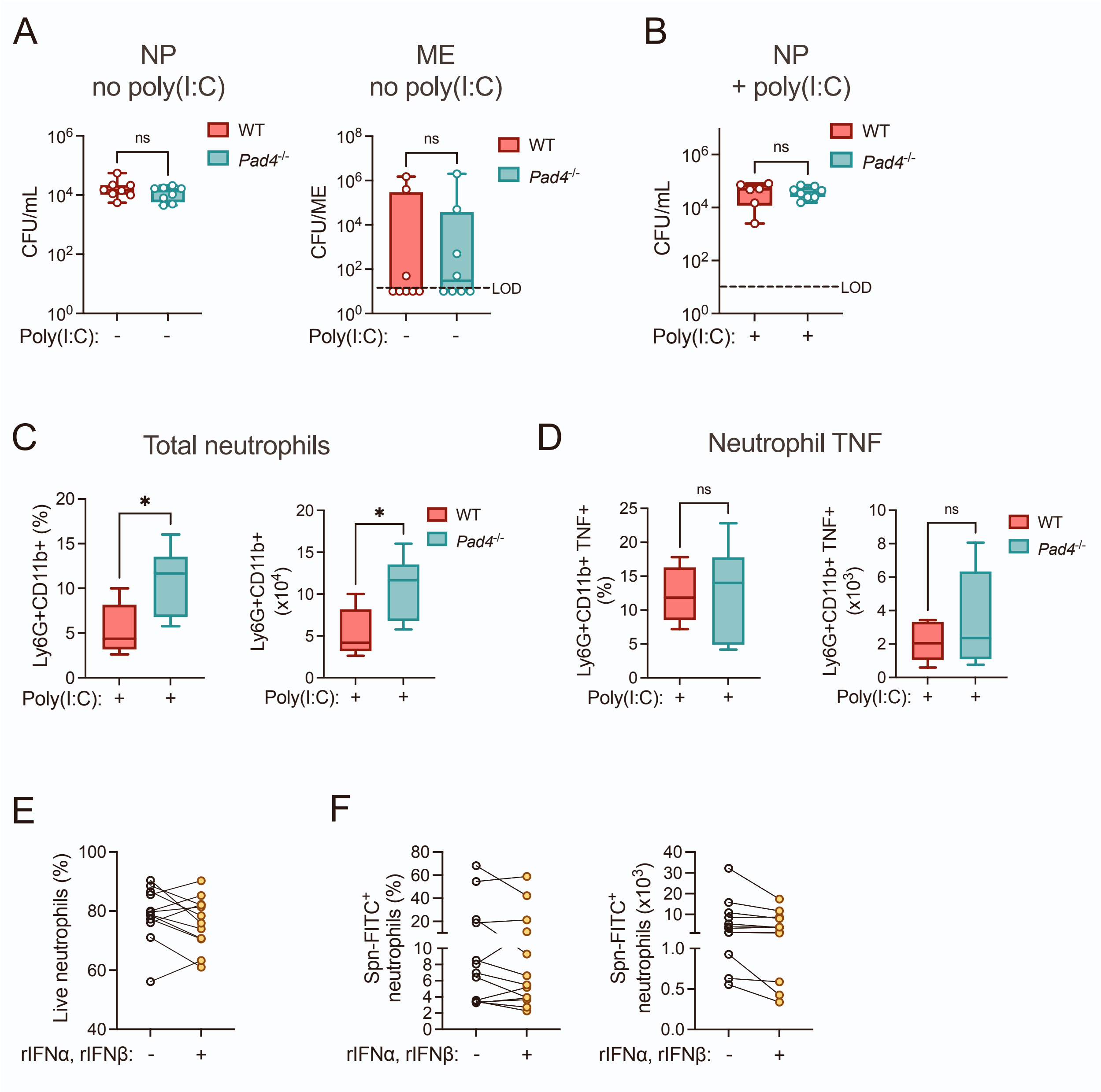

Supplementary Figure 6 (related to Figure 7). Impact of PAD4 deficiency and type I IFN exposure on *S. pneumoniae* infection and neutrophil activation. (A) Burden of *S. pneumoniae* serotype 7F detected in the nasopharynx (NP) and middle ear (ME) in WT or *Pad4*$^{-/-}$ mice at 24 hours post-infection (10^5 CFU/mouse i.n.). (B) Burden of *S. pneumoniae* detected in the nasopharynx of WT or *Pad4*$^{-/-}$ mice at 24 hours post-infection with co-treatment with poly(I:C) (50 µg/mouse i.n.). (C-D) Percent and total number of neutrophils (C) and TNFα$^+$ neutrophils (D) detected by flow cytometry in the middle ear of WT or *Pad4*$^{-/-}$ mice at 24 hours post-infection with *S. pneumoniae* and co-treatment with poly(I:C). (E) Percent of live neutrophils detected by flow cytometry with live/dead staining for neutrophils purified from human blood with or without 1 hour exposure to IFNα2 and IFNβ (1 µg/sample), n=12 subjects. (F) Percent and total number of Spn-FITC$^+$ neutrophils, detected following 1 hour incubation with or without FITC-labeled heat-killed *S. pneumoniae*, for neutrophils purified from human blood with or without 3 hour exposure to IFNα2 and IFNβ (1 µg/sample), n=13 subjects. Data pooled from 3 (A-D) or 5 (E-F) independent experiments. Box boundaries indicate the 25th and 75th percentiles, with a horizontal line representing the median and whiskers indicating minimum and maximum values. LOD = limit of detection. *p<.05, Mann-Whitney U test.
